# Metaproteomic analysis of atmospheric aerosol samples

**DOI:** 10.1007/s00216-016-9747-x

**Published:** 2016-07-13

**Authors:** Fobang Liu, Senchao Lai, Kathrin Reinmuth-Selzle, Jan Frederik Scheel, Janine Fröhlich-Nowoisky, Viviane R. Després, Thorsten Hoffmann, Ulrich Pöschl, Christopher J. Kampf

**Affiliations:** 1 Department of Multiphase Chemistry, Max Planck Institute for Chemistry, Hahn-Meitner-Weg 1, 55128 Mainz, Germany; 2 School of Environment and Energy, South China University of Technology, Higher Education Mega Center, Guangzhou, 510006 People’s Republic of China; 3 Institute of General Botany, Johannes Gutenberg University Mainz, Johannes-von-Müller-Weg 6, 55128 Mainz, Germany; 4 Institute for Inorganic and Analytical Chemistry, Johannes Gutenberg University Mainz, Duesbergweg 10-14, 55128 Mainz, Germany

**Keywords:** Metaproteomics, Atmospheric aerosols, Bioanalytical methods, HPLC, Mass spectrometry

## Abstract

Metaproteomic analysis of air particulate matter provides information about the abundance and properties of bioaerosols in the atmosphere and their influence on climate and public health. We developed and applied efficient methods for the extraction and analysis of proteins from glass fiber filter samples of total, coarse, and fine particulate matter. Size exclusion chromatography was applied to remove matrix components, and sodium dodecyl sulfate polyacrylamide gel electrophoresis (SDS-PAGE) was applied for protein fractionation according to molecular size, followed by in-gel digestion and LC-MS/MS analysis of peptides using a hybrid Quadrupole-Orbitrap MS. Maxquant software and the Swiss-Prot database were used for protein identification. In samples collected at a suburban location in central Europe, we found proteins that originated mainly from plants, fungi, and bacteria, which constitute a major fraction of primary biological aerosol particles (PBAP) in the atmosphere. Allergenic proteins were found in coarse and fine particle samples, and indications for atmospheric degradation of proteins were observed.

**Electronic supplementary material:**

The online version of this article (doi:10.1007/s00216-016-9747-x) contains supplementary material, which is available to authorized users.

**Graphical abstract:**
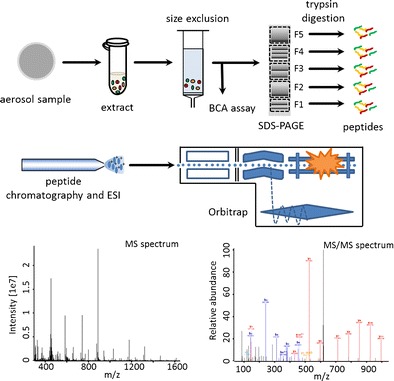
Workflow for the metaproteomic analysis of atmospheric aerosol samples

## Introduction

Primary biological aerosol particles (PBAP) including bacteria, fungal spores, pollen, biogenic polymers, and others like plant or animal fragments, are ubiquitous components of the atmospheric aerosol [1–3]. They likely have an influence on clouds and precipitation [4, 5] and have been linked to many adverse health effects such as infectious, respiratory, and allergic diseases [6–8]. Proteins, contained in PBAP from different sources and with distinct properties, are also known to influence atmospheric microphysics and public health [9–11].

Proteins can be found in coarse mode particles (>2.5 μm aerodynamic diameter) as well as in fine mode particles (<2.5 μm) [12]. It has been shown that bacteria are most frequently observed in ∼2–4 μm particles [13, 14], fungal spores in the range of 2–10 μm [15, 16], pollen grains between 10 and 100 μm [1], and smaller pollen compartments, such as pollen cytoplasmic granules (PCGs; subcellular compartments) released from the rupture of pollen grains due to high humidity and moisture, are in the range of 30 nm to 4 μm [17, 18]. Proteinaceous material in different size modes of atmospheric aerosols have different penetration depths into the human respiratory tract, i.e., fine mode particles are able to pass through the upper respiratory tract and deposit in the small airway and alveoli [19], thus affecting potential health impacts.

Proteins in aerosol particles have been suggested to be good tracers for PBAP in the atmosphere [20]. Many studies have focused on the measurement of total protein content in airborne particles using biological assays, e.g., bicinchoninic acid (BCA) assay, nano-orange, and Bradford assay and found proteins to account for up to 5 % of particles in mass concentration [21–23]. Some specific proteins, mostly allergens, have been investigated using immunoassays, such as enzyme-linked immunosorbent assay (ELISA) or Western blot, etc. For example, Buters et al. [24] determined the major birch pollen allergen Bet v 1 in ambient aerosols of different size fractions with an allergen-specific ELISA. Miyajima et al. [25] developed a fiber-optic chemifluorescence immunoassay for the detection of the airborne major dust mite allergen Der f 1. Although these immunoassays have the advantage of low detection limits and can quantify the targeted proteins, the antigen specificity of these assays limits their use in metaproteomic analysis of ambient aerosol particles. The term metaproteomics has been proposed for the characterization of the entire protein complement of environmental samples at a given point in time [26, 27]. Mass spectrometry-based metaproteomics has been successfully applied in studies of soils, lake sediments, and marine environments [28–31]. With regard to atmospheric aerosols, bioaerosol mass spectrometry has been used for the rapid identification of individual aerosolized microbial particles [32, 33]. Moreover, metaproteomic analysis has recently been applied to soils in Asian desert dust storm deposition regions [34, 35].

**Fig. 1:**
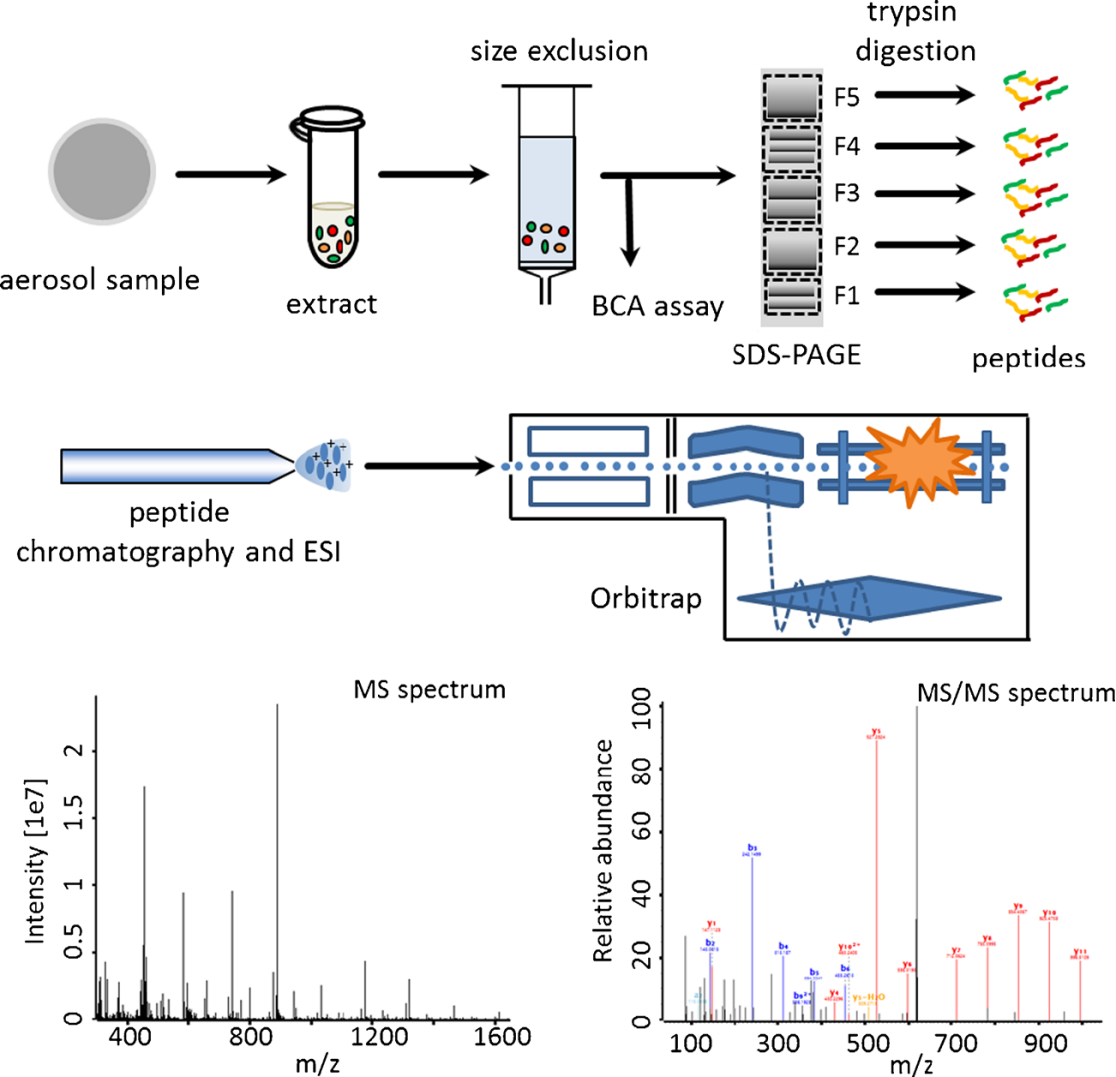
Schematic overview of the developed method for the metaproteomic analysis of atmospheric aerosol samples. Aerosol filter samples were extracted and subjected to size exclusion chromatography to remove sample matrix before BCA assay and SDS-PAGE analysis. Five molecular size fractions were excised from SDS-PAGE gels and in-gel digested before nano-LC-MS/MS with a Hybrid Quadrupole-Orbitrap mass spectrometer. Proteins were identified using the MaxQuant software for database searches against the Swiss-Prot database

In this study, we develop a method to characterize proteins from atmospheric aerosol samples using a mass spectrometry-based metaproteomics approach, providing information about the taxonomic composition of bioaerosols. To our knowledge, this approach has previously not been established and applied for atmospheric aerosol samples. The critical step for protein identification is to efficiently extract proteins from the air filter samples. Besides considering the differences in protein properties such as solubility, also interactions between proteins, particles, and filter material need to be overcome by the extraction method. We evaluated the effects of soot particles and ammonium sulfate on protein recovery during filter extraction using BCA assays and sodium dodecyl sulfate polyacrylamide gel electrophoresis (SDS-PAGE). Furthermore, aerosol samples of different particle size fractions (total suspended, fine, and coarse particles) were analyzed using nano-HPLC coupled with a Hybrid Quadrupole-Orbitrap mass spectrometer after in-gel digestion. The method developed in this study allows for the characterization of aerosol proteins, simultaneously yielding insights into atmospheric protein transformation processes. A schematic overview of the analytical procedure for protein identification in ambient aerosol samples is shown in Fig. 1.

## Materials and methods

### Reagents

Bovine serum albumin (BSA; A5611), phosphate-buffered saline tablet (PBS; P4417), glycine (G7126), β-mercaptoethanol (M-6250), dithiothreitol (DTT; D5545), iodoacetamide (IAM; I6125), acetonitrile (ACN; 34967), ammonium bicarbonate (A6141), and trypsin from porcine pancreas (T6567) were supplied by Sigma-Aldrich (Germany). Ten times Tris/glycine/SDS (161-0732) and two times Lammli sample buffer (161-0737) were from Bio-Rad Laboratories (USA). Trifluoroacetic acid (TFA; 400028) was from Applied Biosystem (UK). Formic acid (28905) and C18 spin tubes (89870) for desalting were obtained from ThermoFisher Scientific (Germany). Diesel particulate matter (SRM 2975) was purchased from the National Institute for Standards and Technology (NIST; USA). Ammonium sulfate (>99 %) was obtained from Acros Organics. Sodium dodecyl sulfate (H5113) was from Promega (USA). Glass fiber filters (type MN 85/90, 406015, Duren, Germany) for sampling of total suspended particles (TSP) and protein recovery tests during the development of the extraction method were purchased from Macherey-Nagel (Germany). A second set of glass fiber filters (type A/A, 102-mm diameter) for sampling coarse and fine particles was obtained from Pall Corporation (UK). High-purity water (18.2 MΩ m) was taken from an ELGA LabWater system (PURELAB Ultra, ELGA LabWater Global Operations, UK) and autoclaved before use if not specified otherwise.

### Aerosol sampling

Aerosol samples were collected at the roof of the Max Planck Institute for Chemistry (MPIC; Mainz, Germany) in 2010 and 2015; the sampling period was generally 7 days. Sampling details are provided in Fröhlich-Nowoisky et al. [15]. Briefly, coarse and fine aerosol particles were collected onto a pair of glass fiber filters (prebaked at 500 °C overnight) by a self-built high-volume dichotomous sampler [36] operated at 300 L/min. Coarse particles with aerodynamic diameters larger than the cutoff diameter (≈3 μm) were collected through a virtual impactor operated in line with the inlet (≈30 L/min), and fine particles with aerodynamic diameters smaller than the cutoff were collected from the main gas flow perpendicular to the inlet (≈270 L/min). As a result of the air flow design of the virtual impactor, 10 % of the fine particles are collected on the coarse particle fraction. Furthermore, TSP samples were collected on 150 mm glass fiber filters (baked overnight at 290 °C) using a self-standing high-volume sampler (Digitel DHA-80) operated at 100 L/min. A list of all investigated air filter samples is given in Electronic supplementary material (ESM) 1 Table S1. The loaded samples were stored in decontaminated aluminum foil bags at −80 °C. To detect possible contaminations from the samplers and sample handling, blank samples were taken as well. Blank sample filters were mounted in the sampler like for regular sampling, but the pump was turned on only for up to 30 s.

### Protein extraction

Figure S1 in ESM 1 illustrates the extraction method development. The effects of the vial material, the extraction solvent and technique, as well as the enrichment method on protein recovery were investigated. In these experiments, 200 μg of BSA dissolved in 100 μL H_2_O were spiked on prebaked filters. The parameters of interest were varied individually, while keeping the remaining parameters constant (see ESM 1 for details). The corresponding effects were evaluated by BSA recovery obtained by BCA assay, which has been widely applied for the measurement of total protein concentration in ambient aerosol samples [22, 37], as outlined in “Bicinchoninic acid assay”. All spiking experiments were performed in triplicate.

The optimized extraction method (discussed in “Development of extraction method”) was applied to aerosol filter samples. Briefly, filter aliquots (∼40 cm^2^) were cut out from the whole filter and extracted twice with 2.0 mL 1× Tris/Gly/SDS buffer in a 15-mL polypropylene (PP) vial by sonication (frequency, 35 kHz; Bandelin, Sonorex Super 10P, Germany) for 1 h. It should be noted that low protein binding microcentrifuge tubes (525-0134, VWR International, Germany) were used in following steps in order to minimize protein loss. After the first extraction, the extract was centrifuged (15,000 rpm, 15 min) and the supernatant was collected before extracting the filter material the second time. Subsequently, the supernatants were lyophilized separately (Christ Alpha 2-4 LD, Germany). The dried residues were resuspended in 500 μL H_2_O and subjected to size exclusion chromatography (28-9180-08, PD Minitrap™ G-25, exclusion limit 5 kDa, GE Healthcare, Germany) according to the supplier’s instruction, before BCA assay and SDS-PAGE analysis. Also, blank filter samples (see “Aerosol sampling”) were treated in the same way.

### Assessment of matrix interferences on BCA assay and SDS-PAGE silver staining

The effects of ammonium sulfate and soot particles interfering with protein concentration determination by BCA assay were investigated. Experiments were conducted in triplicate using aliquots of 26 mg ammonium sulfate, 0.4 mg soot with or without spiking BSA solution (final concentration 250 mg/L) in 500 μL Tris/Gly/SDS buffer, representing the estimated mass of ammonium sulfate and soot collected on the ambient filter samples based on a study by Poulain et al. [38], and the average protein concentration on our filter samples as determined by BCA assay. The mixture was sonicated for 1 h and afterwards centrifuged (15,000 rpm) for 15 min. The supernatant (450 μL) was pipetted into a size exclusion column (PD Minitrap™ G-25) while a 50-μL aliquot was kept as the sample before size exclusion treatment. Both samples, before and after size exclusion treatment, were analyzed by BCA assay.

In addition, 0.4 mg soot samples with or without spiking BSA (200 ng) in 500 μL Tris/Gly/SDS buffer were used to investigate the effect of soot on SDS-PAGE silver staining. The same procedures of sonication and size exclusion treatment were performed as described above. Afterwards, the eluate was lyophilized and resuspended in 40 μL 1× Lammli sample buffer for SDS-PAGE and silver staining, as detailed in “SDS-PAGE and in-gel digestion”.

### Bicinchoninic acid assay

The protein concentrations of spiked BSA and ambient aerosol filter sample extracts were determined with the BCA assay (BCA1-1 KT, Sigma-Aldrich). In brief, the assay was performed in 96-well microplates and calibrated with solutions of BSA dissolved in the corresponding extractants. Volumes of 10 μL of standard and sample solutions, respectively, were pipetted into the microwells (three wells per sample solution), and 200 μL freshly prepared working reagent was added. The microplate was incubated at 60 °C for 15 min, and then cooled to room temperature (∼22 °C). The absorbance was measured on a microplate photometer (Thermo Scientific Multiskan EX) at 560 nm. Prebaked blank filters and sample handling blanks were assayed according to the same procedure, and results were used to correct laboratory and ambient filter results for blank values.

### SDS-PAGE and in-gel digestion

SDS-PAGE was performed using a 4 to 20 % gradient Mini-PROTEAN® TGX™ Gel (456-1093, Bio-Rad, USA). Briefly, after lyophilization, the ambient filter sample extracts were resuspended in 40 μL 1× Lammli sample buffer containing 2.5 % β-mercaptoethanol, then incubated at 95 °C in a thermomixer (Thermomixer Comfort, Eppendorf, Germany) for 5 min prior to SDS-PAGE separation. A molecular weight marker (Precision Plus Protein Unstained Standards, 161-0363, Bio-Rad, USA) was used for molecular weight scale calibration. Gels were run at a constant voltage of 110 V and silver-stained with a Pierce Silver Stain for Mass Spectrometry kit (24600, ThermoFisher Scientific, USA) according to the supplier’s instruction. Subsequently, the gels were scanned on a ChemiDoc MP Imaging system using the Image Lab software (version 4.1, Bio-Rad).

The gels were cut into five fractions (F1-F5) as illustrated in Fig. 3, corresponding to molecular weights of ∼10–15 kDa (F1), ∼15–25 kDa (F2), ∼25–50 kDa (F3), ∼50–100 kDa (F4), and ∼100–250 kDa (F5) for in-gel digestion. The excised pieces were destained using the reagents and procedure provided in the Pierce Silver Stain for Mass Spectrometry kit. The following in-gel digestion was conducted according to the protocol of Shevchenko et al. [39]. Briefly, 10 mM DTT was applied at 56 °C for reduction of disulfide bonds and 55 mM IAM at room temperature in the dark for alkylation of cysteine residues. Trypsin digestion was performed at 37 °C overnight. Typically, 200 μL or more DTT, IAM, and trypsin solution were added to completely cover the gel pieces in the corresponding step, depending on the volume of gel matrix. After digestion, peptides were extracted from the gel pieces by adding 400 μL 5 % formic acid/ACN (*v*/*v*) and incubating for 15 min at 37 °C. Subsequently, the supernatants were collected and dried down by a SpeedVac concentrator (Christ RVC 2-25, Germany). The dried extracts were dissolved in 100 μL 5 % ACN in H_2_O with 0.5 % TFA and desalted with conditioned C18 spin tubes according to the manufacturer’s instructions. Finally, the tryptic peptides were eluted using 20 μL 50 % ACN in H_2_O with 0.1 % formic acid for MS analysis.

### Nano-LC-MS/MS analysis

Peptide mixtures were analyzed with a Thermo Q Exactive Plus Hybrid Quadrupole-Orbitrap mass spectrometer coupled to an EASY nLC 1000 uHPLC system. Self-packed NewObjective silica tip columns (25 cm length, 75 μm inner diameter) packed with C18 stationary phase material (ReproSil-Pur 120 C18-AQ 1.9, 120 Å pore size, 1.9 μm particle size, Dr. Maisch) were used for peptide separation. The column was operated in a column oven at 35 °C to reduce back pressure and coupled to a nano-electrospray ion source [40]. Eluents were H_2_O with 0.1 % formic acid (buffer A) and 80 % ACN in H_2_O with 0.1 % formic acid (buffer B). Peptides were eluted with a linear gradient from 2 to 5 % buffer B for 2 min, 5 to 40 % B for 19 min, 40 to 95 % B for 4 min, and 95 % B for 5 min at a flow rate of 225 nL/min. Then the mobile phase was reset to initial condition within 4 min and equilibrated for 4 min before the next run. The sample injection volume was 9 μL. The Q Exactive Plus Oribtrap was operated in a HCD Top 10 mode with dynamic selection of the ten most intense peaks from each survey scan (*m*/*z* 300–1650) with collision energy of 25 eV for fragmentation. The resolution for full scan (*m*/*z* 300–1650) was 70,000 and 17,500 for MS/MS scan. Dynamic exclusion time was 20 s.

Database searches were performed with Maxquant (version 1.4.1.2, http://www.maxquant.org/) against the database Swiss-Prot (release 2013_08, www.uniprot.org). Trypsin/P was specified as a cleavage enzyme. Carbamidomethyl (C) was set as a fixed modification. Variable modifications were acetyl (protein N-term) and oxidation (methionine (M)). Initial peptide mass tolerance was set to 20 ppm, and fragment mass tolerance was set to 4.5 ppm. Two missed cleavages were allowed, and the minimum peptide length was seven amino acids. The maximum false-discovery rate (FDR) was set to 0.01 for both the peptides and proteins. The maximal posterior error probability (PEP), which is the individual probability of each peptide to be a false hit considering identification score and peptide length, was set to 0.1. Only proteins with a minimum of two identified peptides, one of which needs to be unique, and without simultaneous detection in blank and wash samples were regarded as positively identified.

## Results and discussion

### Development of extraction method

The effects of vial materials, extraction solvents and techniques, as well as enrichment methods on protein recovery from spiked filter samples were investigated (for details, see “Protein extraction”; Fig. S1 in ESM 1). The presented extraction method is primarily aimed at proteins that are already released or easily extractable from pollen, fungal spores, bacteria, and other cells and cellular fragments in the primary biological fraction of air particulate matter.

We first compared the influence of vial materials used for extraction, i.e., PP and glass, on BSA recovery from glass fiber filters. No significant difference in BSA recovery was observed (Δ_recovery_ ∼1 %). Polypropylene vials were selected for further method development steps. Physical extraction methods tested were sonication and stirring. Sonication and stirring were both carried out for 1 h at room temperature. Protein recoveries of sonicated samples were 13 % higher than of stirred samples. Sample enrichment methods tested were freeze drying and protein precipitation using trichloroacetic acid (TCA). Protein recovery of freeze drying was 22 % higher compared with TCA precipitation. Trichloroacetic acid precipitation is efficient for protein separation from sample matrix but lower protein recoveries were obtained. Thus, for maximum protein recovery, freeze drying was used for protein enrichment. It should be noted that a commercial kit for protein extraction from soils (NoviPure® Soil Protein Extraction Kit, Mo-Bio) was also tested but showed a comparatively low recovery (8.5 ± 3.6 %, data not shown) for BSA spiked on test filters. Further tests and procedure optimizations for extraction methods aiming to extract proteins also from intact cells collected on air filter samples, including lysis methods, are required and shall be pursued in follow-up studies.

**Fig. 2:**
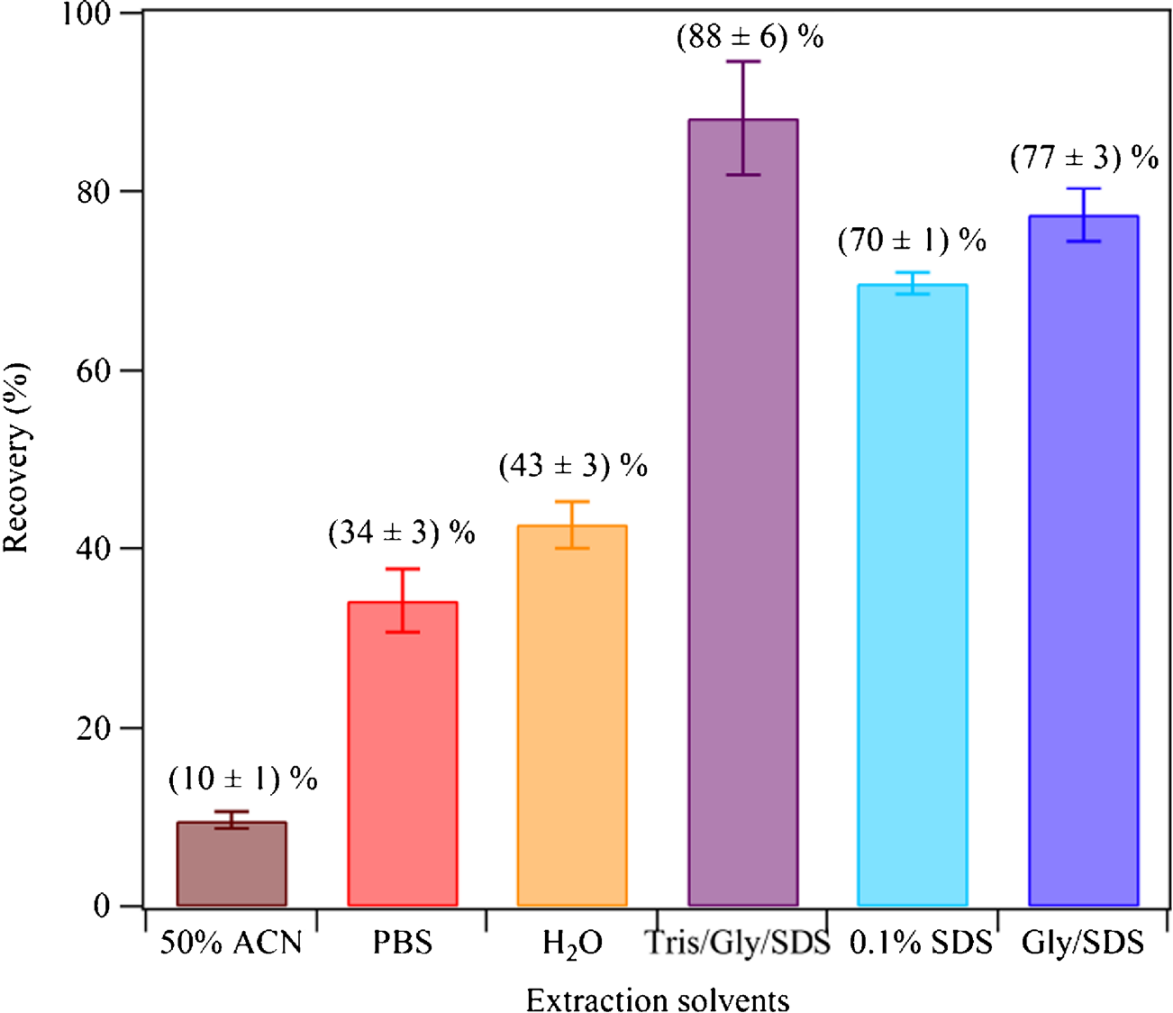
Protein recoveries obtained for different extraction solvents used for the extraction of test filters spiked with 200 μg BSA. The filter extraction procedure and solvents are detailed in “Protein extraction”

The comparison of extraction solvents was performed among H_2_O (as reference), 50 % ACN in H_2_O (common extraction solvent for organic aerosol constituents), and aqueous buffer solutions commonly used in aerosol protein extraction (PBS) and biological research (PBS and Tris/Gly/SDS). The highest protein recovery (88 ± 6 %) was observed for Tris/Gly/SDS buffer (25 mM Tris, 192 mM glycine, 0.1 % SDS in aqueous solution), followed by Gly/SDS (192 mM glycine and 0.1 % SDS in aqueous solution), 0.1 % (*w*/*v*) SDS in H_2_O, H_2_O, 10 % PBS in H_2_O, and 50 % ACN in H_2_O, respectively, as shown in Fig. 2. Sodium dodecyl sulfate (SDS), as an anionic detergent, can denature secondary and non-disulfide-linked tertiary structures of proteins and therefore facilitates the solubilization of otherwise water-insoluble proteins as well as water-soluble proteins. Watanabe et al. [41] reported that the amount of protein extracted from food increased 10- to 100-fold when the extraction solvent contained SDS and β-ME and assumed that SDS helps solubilize proteins by disrupting most of their non-covalent bonds. Indeed, all extraction solvents containing SDS resulted in a high protein recovery. Therefore, Tris/Gly/SDS buffer was selected to enable extraction of water-soluble and water-insoluble proteins and to minimize other potential non-covalent interactions between proteins and components (e.g., soot, dust) of ambient aerosol samples and the filter material.

In summary, in the optimized method, samples were sonicated using Tris/Gly/SDS buffer as the extraction solvent, followed by freeze drying of the obtained extracts for sample enrichment.

### BCA assay and SDS-PAGE silver staining analysis of aerosol samples—interferences caused by ammonium sulfate and soot particles

**Fig. 3:**
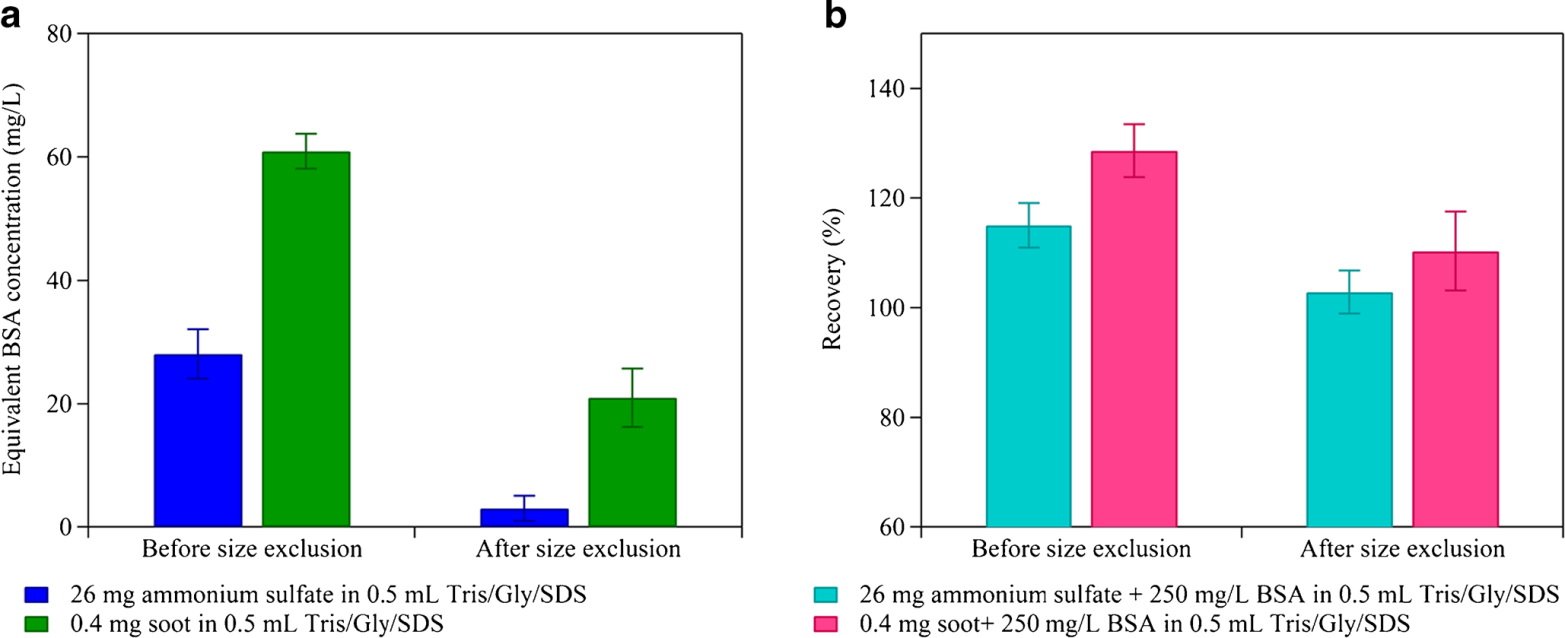
Influence of soot particles and ammonium sulfate on total protein content analysis by BCA assay: **a** equivalent BSA concentration of soot particle and ammonium sulfate standards in Tris/Gly/SDS buffer before and after size exclusion; **b** observed BSA recovery for soot + BSA and ammonium sulfate + BSA mixtures before and after size exclusion

Previous studies have shown that aerosol components such as ammonium sulfate and humic-like substances (HULIS) may hamper protein determination by protein quantitation kits [42]. They found that protein concentrations measured by the protein quantitation kit (nano-orange assay) were six times higher than the concentrations determined by hydrolysis of proteinaceous material and concluded that the discrepancy could be caused by matrix interferences. In addition, also soot particles, which are mostly present in the fine fraction of atmospheric aerosols, may cause interferences in protein concentration determination by protein quantitation kits. Here, we estimate the effects of ammonium sulfate and soot on protein concentration determination by BCA assay (details in “Assessment of matrix interferences on BCA assay and SDS-PAGE silver staining”). Figure 3a illustrates that ammonium sulfate and soot are causing signals in the BCA assay (signals were converted into equivalent BSA concentrations) and thus the calculated recovery of BSA was >100 % in Fig. 3b, when ammonium sulfate or soot were present in the protein solution.

Low molecular weight interfering substances, i.e., ammonium sulfate, can be efficiently removed by size exclusion chromatography (SEC), as suggested by Franze et al. [22] and illustrated in Fig. 3. The BCA assay signal caused by ammonium sulfate is reduced by around one order of magnitude after SEC, bringing the observed recovery of the BSA/ammonium sulfate mixture close to 100 %. Also for soot particles, a threefold reduction in the BSA equivalent concentration was observed after SEC and >65 % of the interference in the mixed BSA/soot sample could be removed. BCA assay analysis of ambient aerosol samples also show a reduction of BSA equivalent protein concentration of ∼60–90 % after SEC (see Fig. S2 in ESM 1). This reduction in the observed signal may either be caused by an over determination of the protein content in the presence of the aforementioned interferences or by the removal of proteins attached to soot particles. A combination with other protein purification techniques, e.g., dialysis or affinity chromatography, may further improve protein concentration determination of aerosol samples by BCA assay.

**Fig. 4:**
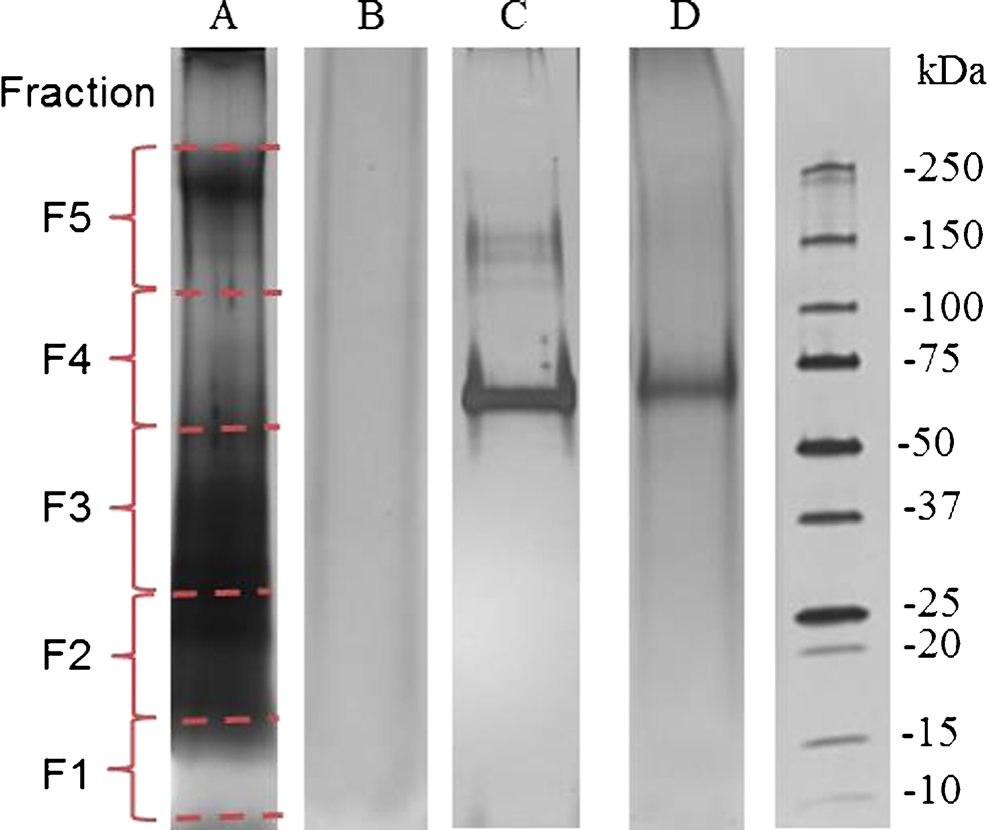
SDS-PAGE of filter extracts after silver staining, BSA and/or soot in Tris/Gly/SDS buffer: *Lane A*, filter sample (Mz02c, TSP); *lane B*, 0.4 mg soot in 500 μL Tris/Gly/SDS buffer; *lane C*, 200 ng BSA in 500 μL Tris/Gly/SDS buffer; *lane D*, 200 ng BSA mixed with 0.4 mg soot in 500 μL Tris/Gly/SDS buffer. *Right lane*, protein molecular weight marker

For SDS-PAGE analysis, no influence of ammonium sulfate was observed, but soot particles were found to affect the appearance of the gel after silver staining. Figure 4 lane A shows the separation of an ambient aerosol (TSP) sample extract after silver staining, whereby no clear bands could be resolved over the strong background. Coomassie-stained SDS-PAGE gels of filter extracts did not show any visible bands (data not shown; EZBlue, G1041, Sigma-Aldrich, Germany, detection sensitivity 5 ng), indicating that overloading of gels is no major issue and silver staining was selected because of its higher sensitivity (<1 ng protein) [43]. Additional experiments were performed to investigate the influence of soot particles on the lane background after silver staining (see Fig. 4; Fig. S3 in ESM 1). The background of lanes showing separations of samples with soot standard (lane B and D in Fig. 4; lane B in Fig. S3 in ESM 1) appears in a darker color after silver staining compared with the background of lanes without the addition of soot (lane C Fig. 4; Fig. S3 in ESM 1). Furthermore, the intensities of the protein bands were weaker in the presence of soot particles (lane D in Fig. 4; lane B in Fig. S3 in ESM 1). However, the location of the BSA monomer band remained unaffected by the soot particles (lanes C and D in Fig. 4). It should be noted that the soot standard used here might have different properties than aged soot in the atmosphere, as soot morphology changes and coatings by organic substances have been observed for atmospherically aged soot particles [44]. Alternative or optimized staining methods shall be investigated in follow-up studies to minimize the effect of soot particles in the staining step.

Soot particles were found to affect both BCA assay and SDS-PAGE analysis of atmospheric aerosol samples and should be considered when reporting the corresponding protein concentrations. Nevertheless, the soot particles did not affect SDS-PAGE protein separation itself, thus in-gel digestion of aerosol sample extracts and subsequent peptide LC-MS/MS have been performed and will be discussed in the next section.

### Protein identification in ambient aerosol samples

Ambient aerosol samples collected in Mainz, Germany, a sampling site in central Europe influenced by urban and rural boundary layer air masses, have been analyzed. A list of the identified proteins and their taxonomic classification is given in ESM 2. The Maxquant output file for the observed peptides of the identified proteins and protein groups, respectively, is provided in ESM 3.

Five, twenty-one, and thirty-three proteins were successfully identified in the coarse, fine, and TSP aerosol sample, respectively. There seems to be a gap in the number of identified proteins from the investigated aerosol samples compared with the metaproteomic analysis of other environmental samples, e.g., soils or sediments [28–31]. The low number of proteins identified in this work might be related to the applied extraction method, for which the aims were outlined above. Also, larger sample sizes (whole filters, longer sampling times) might be used to increase the number of identified proteins, considering the potentially low amounts of individual proteins contributing to the total protein mass analyzed per filter aliquot (~250 µg) due to the diversity of protein sources including various plants, fungi, and bacteria. Furthermore, as will be discussed below, we observed the presence of partly degraded proteins in the aerosol filter sample extracts, which may further hamper protein identification. Note that the higher number of identified proteins in the fine fraction aerosol sample compared with the coarse fraction aerosol sample is likely due to the higher sampling flow rate of the fine fraction aerosol sample. Protein databases (e.g., Swiss-Prot) only provide sequence information for a subset of known proteins [45]. Therefore, only those proteins listed in the databases can be identified, which is particularly important for the identification of fungal and bacterial proteins.

Many database-listed proteins of bacteria and fungi are inferred from homology, i.e., indicating that the existence of a protein is probable because clear orthologs exist in closely related species, while no direct experimental evidence for the existence of these proteins exists on a transcript or protein level. For example, the genome of *Neurospora crassa* (a fungi from the class of Sordariomycetes in the phylum of Ascomycota) has been sequenced due to its use as a model organism in biology [46], providing information about predicted protein-coding sequences. Still, proteins identified to orginate from *N. crassa*, which was also found in air filter samples collected in Mainz in March 2006 using DNA analysis [15], are partly inferred from homology (entries 18, 19, 47, and 48 in ESM 2). For other identified proteins, experimental evidence is available at the transcript level (entries 2 and 20 in ESM 2), while experimental evidence at the protein level is only available for one of them (entry 49 in ESM 2).

Some of the identified proteins are expressed by a variety of organisms with only minor changes in the primary protein structure (i.e., the amino acid sequence of the protein). Thus, the taxonomic level to which identified proteins can be assigned varies depending on the uniqueness of the measured peptides among the database-listed proteins. In most cases kingdom (83 %) and phylum (80 %) level assignments are reasonable. The identified proteins mainly originated from plants (68 % in TSP, 31 % in fine particles), microorganisms (fungi, bacteria, Amoebozoa, etc., 25 % in TSP, 50 % in fine particles) and animals (7 % in TSP, 19 % in fine particles), which is in line with the major categories of PBAP [1]. Notably, in the coarse particle sample one protein has been assigned to a bacterium (*Rhodococcus rhodochrous*) [47], which is used as a soil inoculant in agriculture, while potential assignments of proteins identified in the fine particle and TSP sample to the kingdom of bacteria were not unambiguous on the kingdom level.

Also lower taxonomic ranks down to family and genus level may be assigned, e.g., in the case of the well-studied plant pollen proteins. Here, also seasonal influences are reflected in the identified proteins. Particularly for the TSP sample collected beginning of July 2015, several proteins from different grass (Poaceae) genera were identified, which is in accordance with the main grass flowering period from May to July in central Europe [10]. Grass pollen proteins identified were all allergens from the genera *Lolium*, *Dactylis*, and *Phleum*. Notably, also nine proteins originating from *Glycine max* (soybean), eight having a molecular weight >20 kDa, were identified in the TSP sample. Potentially, the occurrence of these proteins can be attributed to soy unloading and industrial processing by a local manufacturing site producing among others soy oil and soy flour. Soy flour dust is known to contain high levels of proteins with MW >20 kDa [48].

Allergenic proteins were found in TSP, coarse, and fine particle samples. Polcalcin Phl p 7 from common timothy (*Phleum pratense*), which is one of the most abundant sources of airborne grass pollen [10], was identified in the TSP and coarse particle sample, while for example, the major perennial ryegrass (*Lolium perenne*) pollen protein Lol p 5a and the hydrophobic seed protein (Gly m 1), an allergen from soybean (*G. max*), were identified in the TSP and fine particle sample. Allergens associated with different aerosol size fractions can be inhaled and transported to different regions of the respiratory tract depending on their size (i.e., smaller particles can enter deeper into the respiratory tract), and thus have distinct health implications such as allergic asthma [49, 50].

**Fig. 5:**
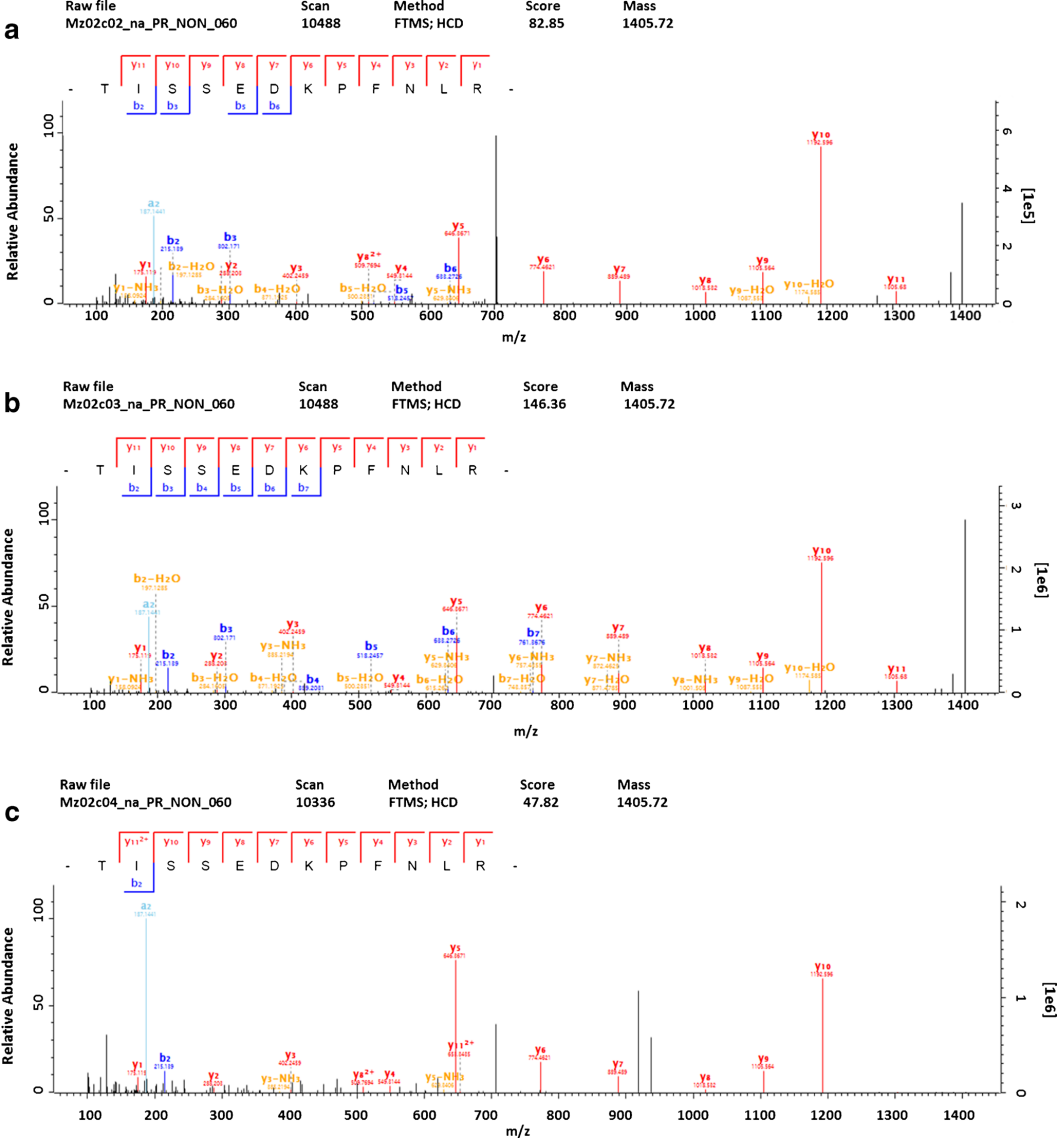
Exemplary MS/MS spectra of the tryptic peptide TISSEDKPFNLR (a unique peptide of Beta-conglycinin, alpha chain from soybean) identified in fraction F2 (**a**), F3 (**b**), and F4 (**c**) of the TSP sample (Mz02c)

The molecular weight-dependent proteomic analysis of aerosol samples showed the presence of protein fragments in the atmospheric aerosol sample extracts. SDS-PAGE gels were divided into five molecular weight fractions (F1-F5, see “Assessment of matrix interferences on BCA assay and SDS-PAGE silver staining”) and some of the identified proteins could be detected in multiple gel fractions (Table 1), i.e., also in fractions corresponding to lower MW than that of the intact protein. For example, beta-conglycinin, alpha chain from soybean (*G. max*), MW 70.3 kDa, was simultaneously identified in fractions F2, F3, and F4 in the TSP sample. Corresponding to its MW, the protein should only be detected in fraction F4. Figure 5 shows exemplary MS/MS spectra of TISSEDKPFNLR, a representative unique peptide of beta-conglycinin, alpha chain, identified in the different MW fractions, respectively. Tandem mass spectra (MS/MS) of other (razor and unique) peptides of beta-conglycinin, alpha chain are shown in Fig. S3 (ESM 1). In general, several processes may lead to the observed protein degradation, including proteolytic degradation during sample preparation and in the environment, as well as degradation by reactive oxygen species (e.g., OH, HO_2_) [51] and acid-catalyzed hydrolysis [52] in the environment. To differentiate between environmental protein degradation and degradation during sample preparation, it is planned to conduct experiments with and without the addition of protease inhibitors [53]. Nevertheless, these first results could motivate studies concerning the fate of proteins in the atmosphere, especially under the rising air pollutant concentrations encountered in the Anthropocene, the present era of steeply increasing human influence on planet Earth [54, 55]. Environmental protein degradation might be a source of peptides, amino acids, amino, and carbonyl compounds in the atmosphere and thus contribute to various atmospheric processes and ecosystem interactions of atmospheric aerosols [56, 57].

**Table 1:** Exemplary results of protein identification in SDS-PAGE molecular size fractions for the TSP and fine particle sample extracts

Sample ID (size range)	Protein name	Family/species	Sum of peptides	Unique peptides	MW (kDa)	(Unique) Peptide counts				
						F1	F2	F3	F4	F5
Mz02c (TSP)	Glycinin G4	Fabaceae/*Glycine max* (soybean)	2	1	63.6	1 (0)	2 (1)	1 (0)	1 (0)	
	Glycinin G1	Fabaceae/*G. max* (soybean)	7	4	55.7	4 (3)	5 (3)	3 (1)	1 (1)	
	Glycinin G2	Fabaceae/*G. max* (soybean)	8	4	54.4	1 (0)	5 (3)	4 (2)		
	Beta-conglycinin, alpha chain	Fabaceae/*G. max* (soybean)	9	6	70.3		2 (2)	9 (5)	4 (4)	
	Beta-conglycinin, alpha’ chain	Fabaceae/*G. max* (soybean)	4	2	74.3			4 (2)		
	Major pollen allergen Lol p 5a	Poaceae/*Lolium perenne* (perennial ryegrass)	6	6	30.9		6 (2)	2 (2)		
344b (<3 μm)	ATP synthase subunit beta	Saccharomycetaceae (yeasts)/–	7	1	54.8	5 (1)	3 (1)	6 (1)	3 (0)	1 (0)
	Elongation factor 2	Saccharomycetaceae (yeasts)/–	3	1	93.2	3 (1)	1 (1)			

Molecular size fractions: F1 (∼10–15 kDa), F2 (∼15–25 kDa), F3 (∼25–50 kDa), F4 (∼50–100 kDa), F5 (∼100–250 kDa)

## Conclusions

Mass spectrometric identification of proteins in atmospheric aerosol samples was carried out after development of a method optimized to extract proteins from air filter samples. Soot particles contained in the aerosol samples were found to interfere with BCA assay analysis, a common technique to measure total protein contents, as well as staining methods, i.e., silver staining, used to visualize SDS-PAGE results. The interference of the soot particles could be minimized by performing size exclusion chromatography of air filter sample extracts.

The metaproteomic analysis presented here allows a first profiling of proteins in atmospheric aerosols. More in-depth analysis of specific post-translational modifications (PTM) of health-relevant proteins (aeroallergens) in the atmosphere (e.g., protein nitration) [58–61], requires specific and efficient enrichment and purification methods, e.g., antibody-based affinity enrichment, which will be addressed in follow-up studies. Furthermore, improvements of protein databases, e.g., by providing proteome information for a larger number of species including fungi and bacteria present in the atmosphere are needed to provide more complete information about the abundance and proportions of different biological kingdoms present in the aerosol metaproteome.

The molecular size-dependent analysis of proteins extracted from the aerosol samples revealed the presence of fragmented proteins in the sample extracts. Such fragments may arise partly from proteolytic degradation during sample preparation and degradation of proteins in the environment, which will be examined in follow-up studies. Environmental protein degradation processes might be of relevance for ecosystem interactions, e.g., nutrient cycling, as well as health implications of protein-containing aerosols due to a potential loss of protein activity upon degradation.

The presented profiles of extractable proteins in atmospheric aerosol particles show that proteins encountered in ambient air particulate matter mainly originate from plants, fungi, and bacteria, which is in line with the major categories of PBAP. Allergenic pollen proteins, e.g., from perennial ryegrass, were found in coarse and fine particles, which can penetrate deep into the lower part of the respiratory tract.

Complementary to antibody or DNA-based methods, the metaproteomic analysis of atmospheric aerosol samples provides a tool to study bioparticles and allergens in air particulate matter. Potential applications include investigations of the spatiotemporal variability of bioaerosol composition and corresponding implications for human health.

## Electronic supplementary material

Below is the link to the electronic supplementary material.

ESM 1 This file contains one table and three figures. Table S1 contains sampling information of the air filter samples. Figure S1 illustrates the development of the extraction method schematically, Fig. S2 shows protein concentrations in air filter samples determined by BCA assay, Fig. S3 provides MS/MS spectra of tryptic peptides of the beta-conglycinin, alpha chain protein identified in the TSP sample. (PDF 1323 kb)

ESM 2 A complete list of accepted identification of proteins/protein groups in ambient aerosol filter samples. The file shows relevant parameters, such as number of peptides (N), sum of razor and unique peptides (O), number of unique peptides (P), number of peptides in respective SDS-PAGE fractions (Q-U), molecular weight (V), sequence coverage (W), and intensity (Y) of the identified protein, PEP score of protein (X, a PEP of 0 is <4.9E−324). Also kingdom (F), phylum (G), class (H), order (I), family (J), genus (K), and species (M) of proteins are summarized in this file. (PDF 330 kb)

ESM 3 This file provides peptide data for the proteins and protein groups identified and listed in Electronic supplementary material 2. (PDF 929 kb)
