# Supplementary material for: Metaproteomic analysis of atmospheric aerosol samples
Source: Anal Bioanal Chem. 2016 Jul 13;408(23):6337–48. doi: 10.1007/s00216-016-9747-x (PMC5009178; doi:10.1007/s00216-016-9747-x)
Supplement: Supplementary file 1 — This file contains one table and three figures. Table S1 contains sampling information of the air filter samples. Figure S1 illustrates the development of the extraction method schematically, Fig. S2 shows protein concentrations in air filter samples determined by BCA assay, Fig. S3 provides MS/MS spectra of tryptic peptides of the beta-conglycinin, alpha chain protein identified in the TSP sample. (PDF 1323 kb) [file 216_2016_9747_MOESM1_ESM.pdf]

Electronic Supplementary Material 1

Metaproteomic analysis of atmospheric aerosol samples

Analytical and Bioanalytical Chemistry

Fobang Liu[1], Senchao Lai[1,2], Kathrin Reinmuth-Selzle[1], Jan Frederik Scheel[1], Janine Fröhlich-Nowoisky[1], Viviane R. Després[3], Thorsten Hoffmann[4], Ulrich Pöschl[1], and Christopher J. Kampf[4,1,*]

[1]Department of Multiphase Chemistry, Max Planck Institute for Chemistry, Hahn-Meitner-Weg 1, 55128 Mainz, Germany

[2]School of Environment and Energy, South China University of Technology, Higher Education Mega Center, Guangzhou 510006, P.R. China

[3]Institute of General Botany, Johannes Gutenberg University Mainz, Johannes-von-Müller-Weg 6, 55128 Mainz, Germany

[4]Institute for Inorganic and Analytical Chemistry, Johannes Gutenberg University Mainz, Duesbergweg 10-14, 55128 Mainz, Germany

[*]Correspondence to Christopher J. Kampf, email: c.kampf@mpic.de, phone: +49 6131 305 6206

Electronic Supplementary 2 - 216_2016_9747_MOESM2_ESM.pdf

Electronic Supplementary 3 - 216_2016_9747_MOESM3_ESM.pdf

Table S1. Overview of all investigated air filter samples in this study.

Sample ID	Size range	Sampling period	Sampled air volume (m^3)	Protein concentration ($\mu g/m^3$)
344a	> 3 µm (coarse)	22/06/2010-29/06/2010	302	0.27 ± 0.01
344b	< 3 µm (fine)	22/06/2010-29/06/2010	2722	0.13 ± 0.01
Mz01a	> 3 µm (coarse)	02/06/2015-09/06/2015	301	0.32 ± 0.02
Mz01b	< 3 µm (fine)	02/06/2015-09/06/2015	2705	0.11 ± 0.01
Mz01c	TSP	02/06/2015-09/06/2015	1002	1.14 ± 0.11
Mz02c	TSP	01/07/2015-07/07/2015	975	1.27 ± 0.03

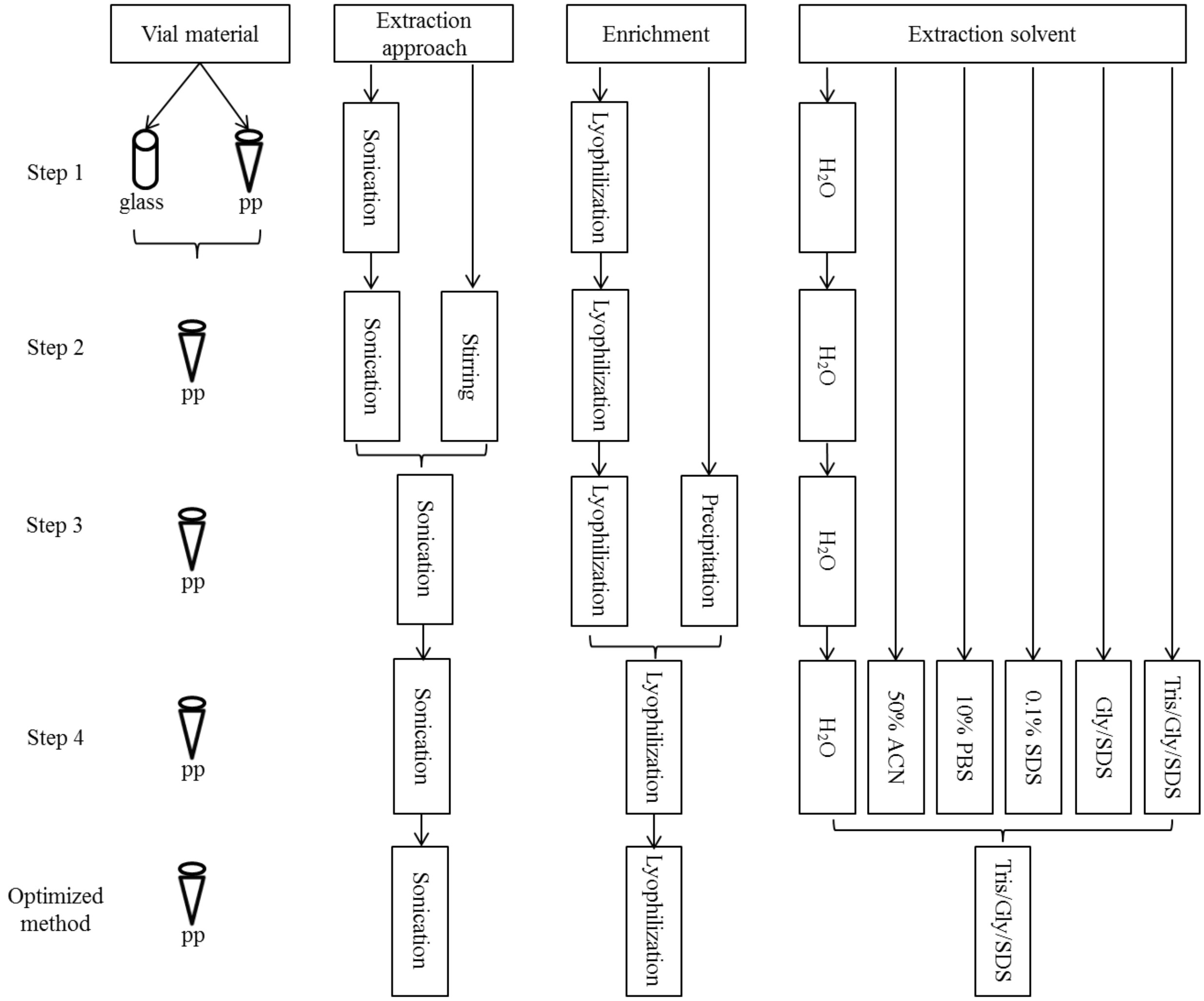

Fig. S1 Scheme of the development of extraction method. Sonication and stirring (step 2) were both conducted twice with 2 mL corresponding solvent for 1h. Precipitation (step 3) was performed with 100% trichloroacetic acid (TCA). Briefly, 0.37 mL 100% TCA was added to each c.a. 1.5 mL extract and incubated at -20°C for 1 h. Then the extract was centrifuged (15,000 rpm, 5 min) and the liquid was removed as much as possible. Subsequently 0.5 mL ice cold acetone was added to wash protein pellets for three times and afterwards the pellets were dried in a hood.

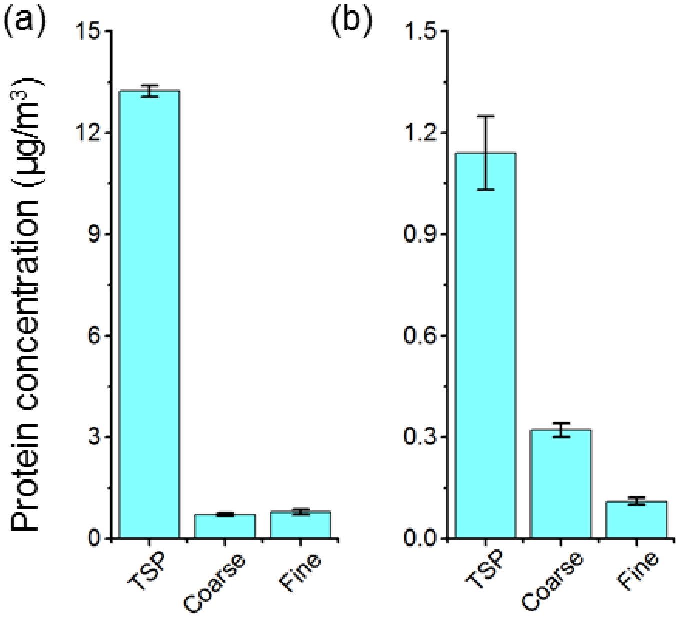

Fig. S2 Protein concentrations before (a) and after (b) size exclusion in ambient TSP (Mz01c), coarse (Mz01a) and fine (Mz01b) fraction particles, sampling in 02/06/2015-09/06/2015.

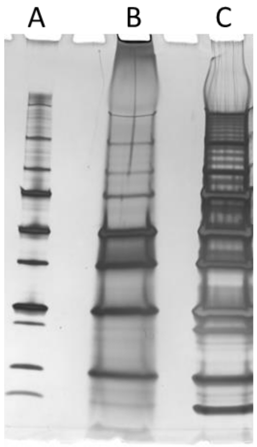

A: 0.5μL MW marker
B: 0.4 mg soot + 2.5 μL MW marker
C: 2.5 μL MW marker

Fig. S3 SDS-PAGE of protein molecular weight (MW) marker after silver staining.

(a)

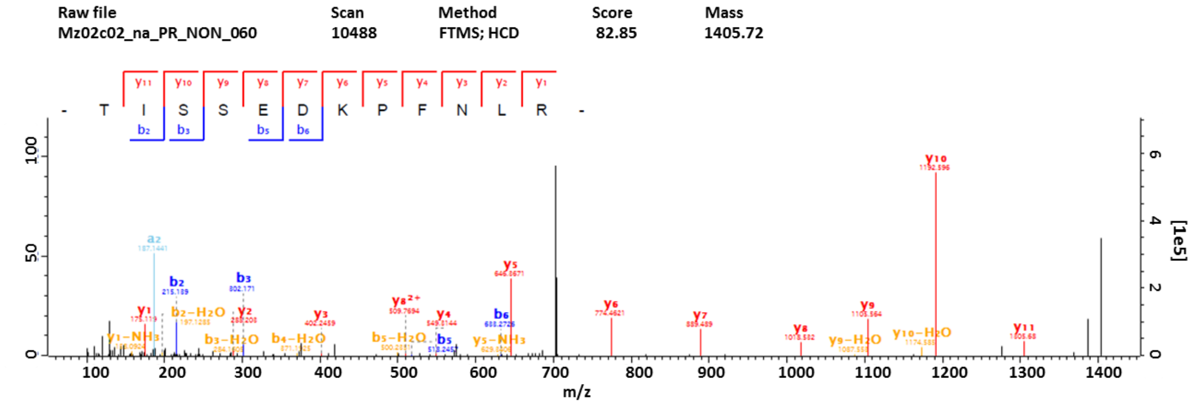
Raw file
Mz02c02_na_PR_NON_060
Scan
10488
Method
FTMS; HCD
Score
82.85
Mass
1405.72
- T I S S E D K P F N L R -
Relative abundance
m/z
[1e5]

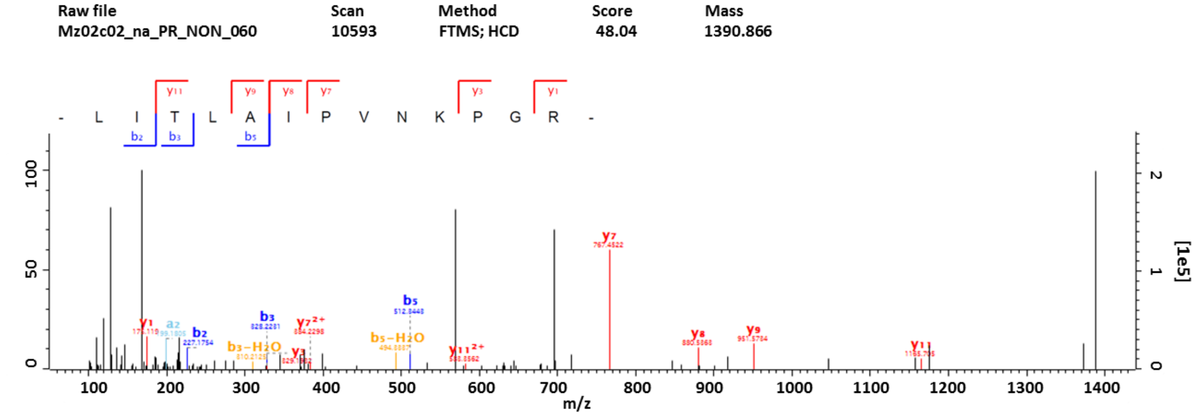
Raw file
Mz02c02_na_PR_NON_060
Scan
10593
Method
FTMS; HCD
Score
48.04
Mass
1390.866
- L I T L A I P V N K P G R -
Relative abundance
m/z
[1e5]

(b)

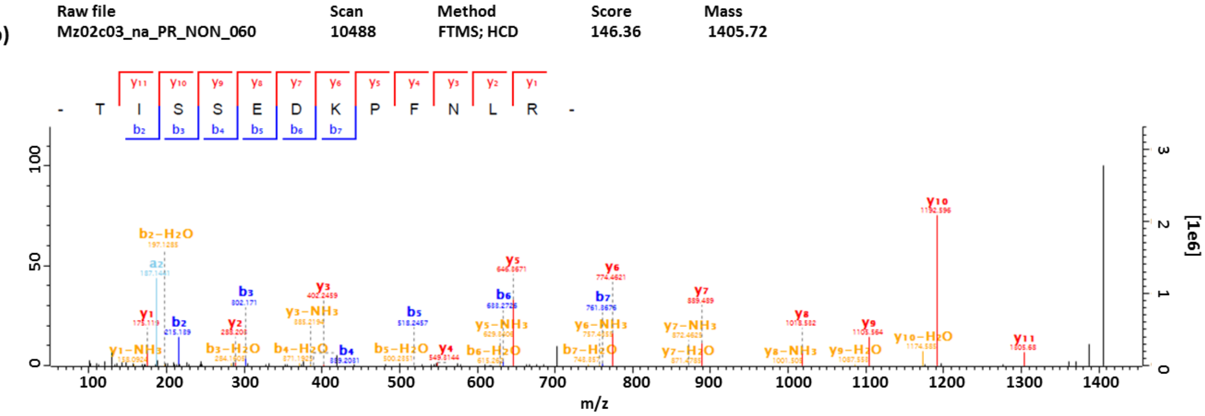
Raw file
Mz02c03_na_PR_NON_060
Scan
10488
Method
FTMS; HCD
Score
146.36
Mass
1405.72
- T I S S E D K P F N L R -
Relative abundance
m/z
[1e6]

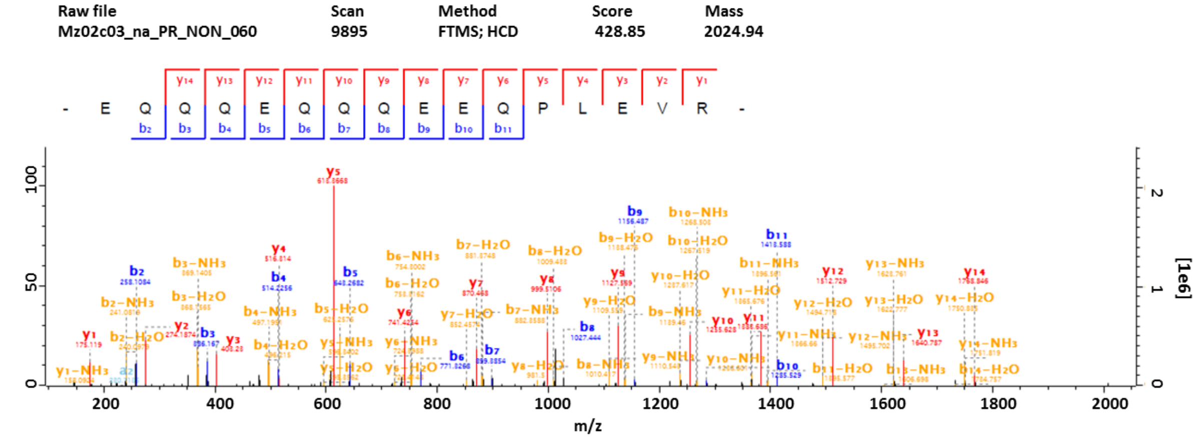
Raw file
Mz02c03_na_PR_NON_060
Scan
9895
Method
FTMS; HCD
Score
428.85
Mass
2024.94
- E Q Q Q E Q Q Q E E Q P L E V R -
Relative abundance
m/z
[1e6]

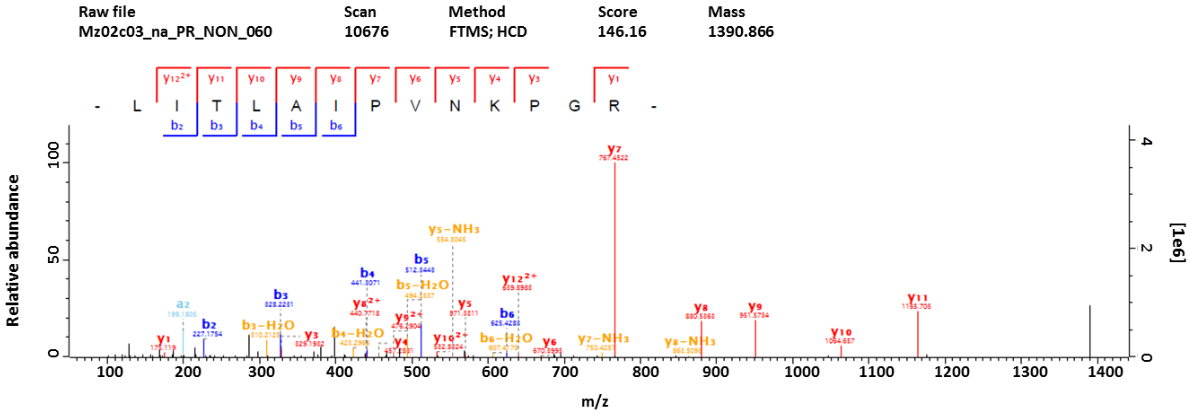
Raw file
Mz02c03_na_PR_NON_060
Scan
10676
Method
FTMS; HCD
Score
146.16
Mass
1390.866
- L I T L A I P V N K P G R -
Relative abundance
m/z
[1e6]

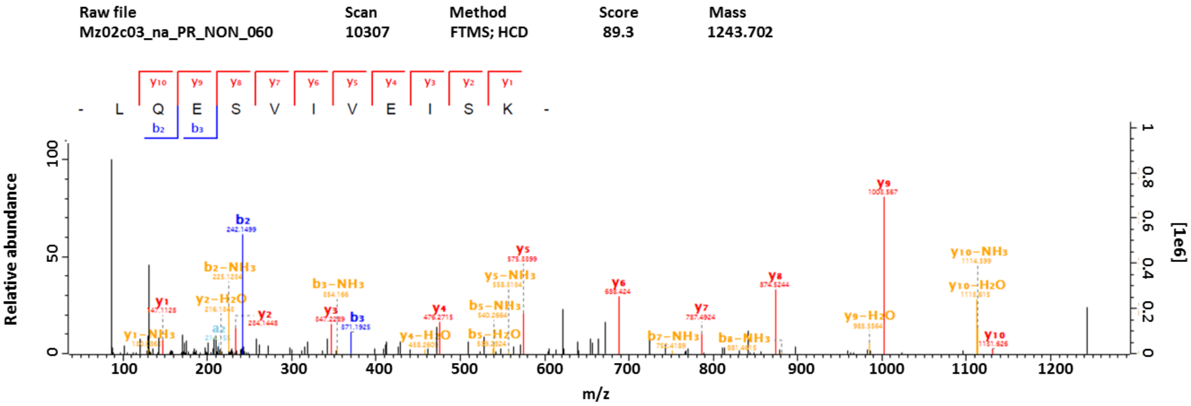
Raw file
Mz02c03_na_PR_NON_060
Scan
10307
Method
FTMS; HCD
Score
89.3
Mass
1243.702
- L Q E S V I V E I S K -
Relative abundance
m/z
[1e6]

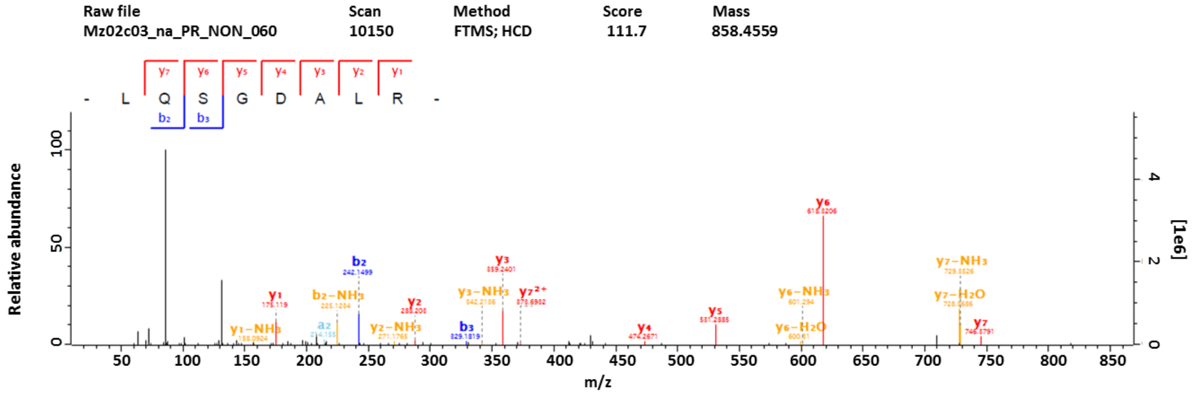
Raw file
Mz02c03_na_PR_NON_060
Scan
10150
Method
FTMS; HCD
Score
111.7
Mass
858.4559
- L Q S G D A L R -
Relative abundance
m/z
[1e6]

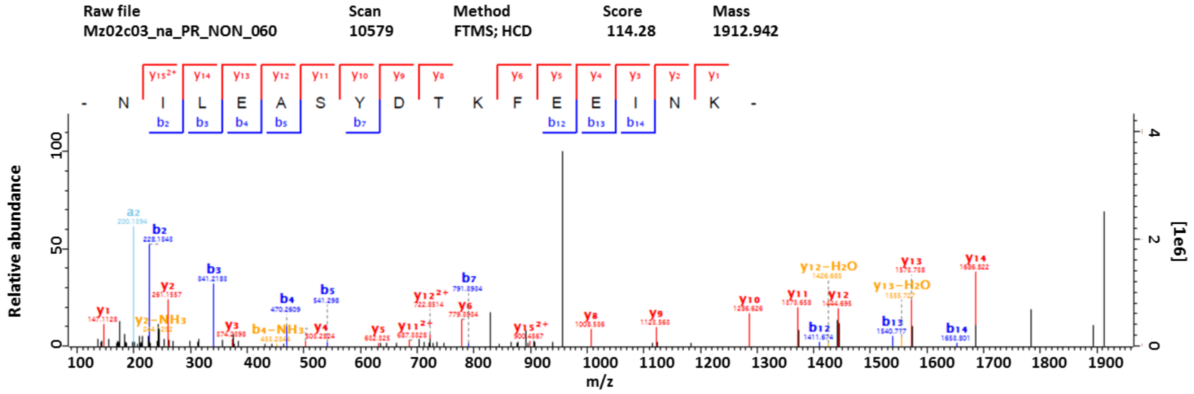
Raw file
Mz02c03_na_PR_NON_060
Scan
10579
Method
FTMS; HCD
Score
114.28
Mass
1912.942
- N I L E A S Y D T K F E E I N K -
Relative abundance
m/z
[1e6]

Raw file	Scan	Method	Score	Mass
Mz02c03_na_PR_NON_060	10004	FTMS; HCD	275.37	2151.023

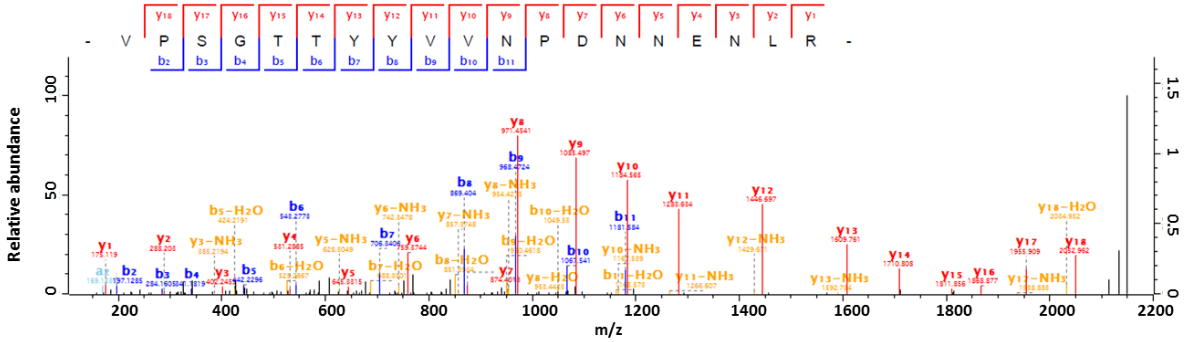

Raw file	Scan	Method	Score	Mass
Mz02c03_na_PR_NON_060	10645	FTMS; HCD	134.56	1617.852

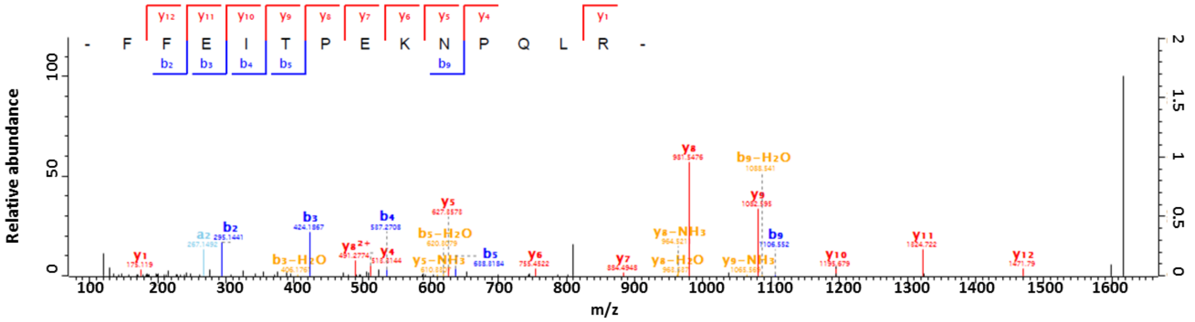

Raw file	Scan	Method	Score	Mass
Mz02c03_na_PR_NON_060	10722	FTMS; HCD	124.07	1533.815

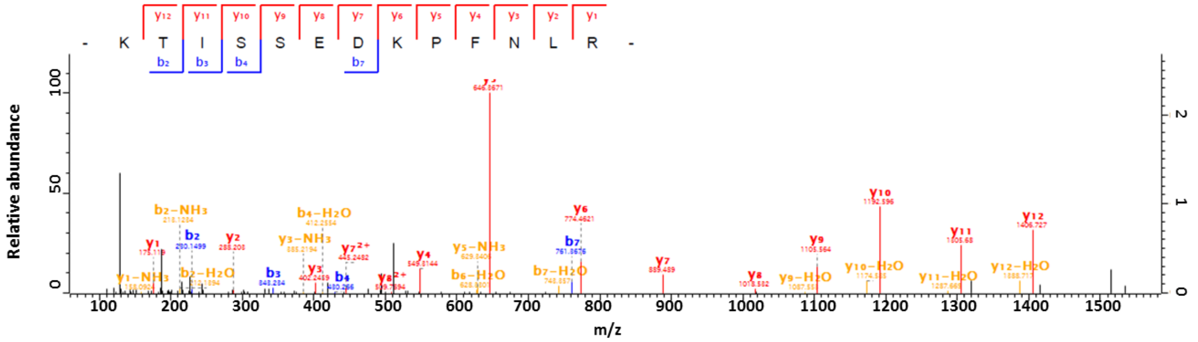

(c)

Raw file	Scan	Method	Score	Mass
Mz02c04_na_PR_NON_060	10336	FTMS; HCD	47.82	1405.72

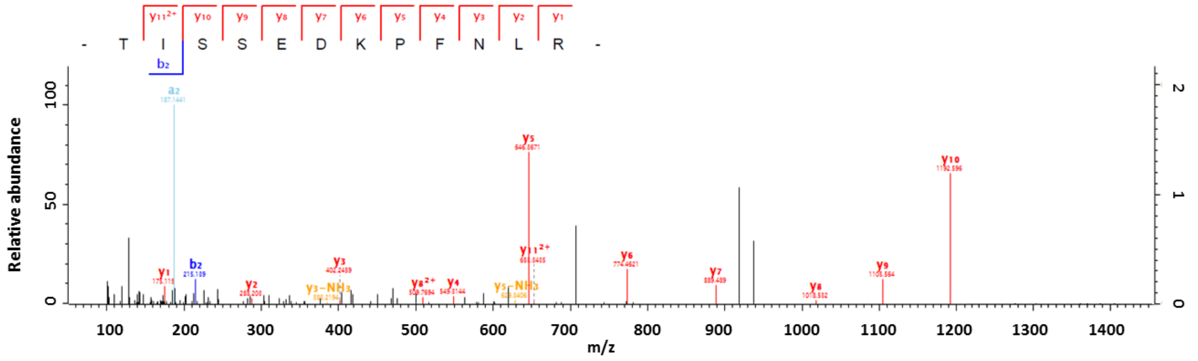

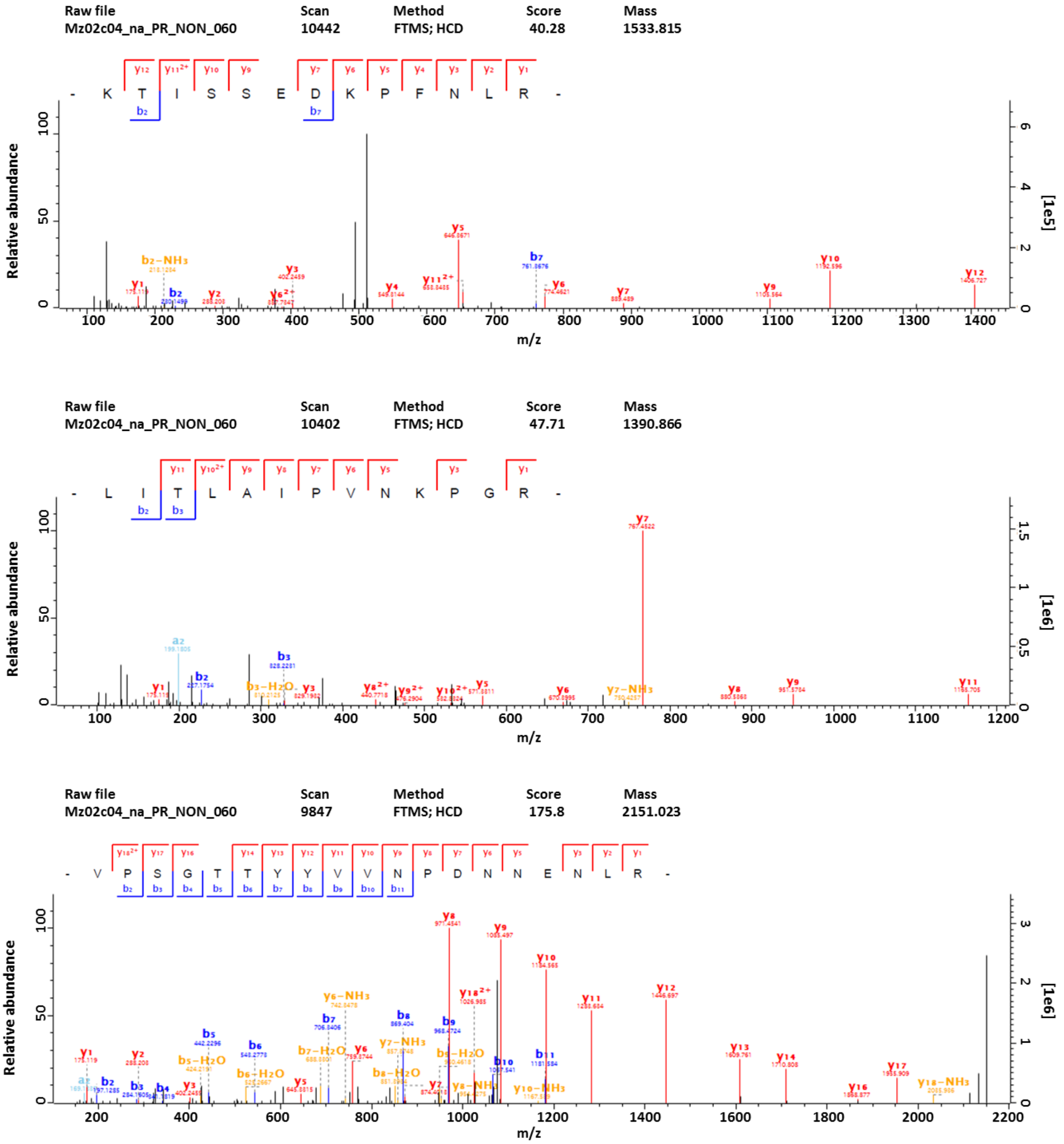

Fig. S4 MS/MS spectra of tryptic peptides of Beta-conglycinin, alpha chain in fraction F2 ((a), two unique peptides), F3 ((b), five unique peptides and four razor peptides) and F4 ((c), four unique peptides) in the TSP (Mz02c) sample.
