# Supplementary material for: Metaproteomic analysis of atmospheric aerosol samples
Source: Anal Bioanal Chem. 2016 Jul 13;408(23):6337–48. doi: 10.1007/s00216-016-9747-x (PMC5009178; doi:10.1007/s00216-016-9747-x)
Supplement: Supplementary file 2 — A complete list of accepted identification of proteins/protein groups in ambient aerosol filter samples. The file shows relevant parameters, such as number of peptides (N), sum of razor and unique peptides (O), number of unique peptides (P), number of peptides in respective SDS-PAGE fractions (Q-U), molecular weight (V), sequence coverage (W), and intensity (Y) of the identified protein, PEP score of protein (X, a PEP of 0 is <4.9E−324). Also kingdom (F), phylum (G), class (H), order (I), family (J), genus (K), and species (M) of proteins are summarized in this file. (PDF 330 kb) [file 216_2016_9747_MOESM2_ESM.pdf]

Metaproteomic analysis of atmospheric aerosol samples (Electronic Supplementary Material 2)

Analytical and Bioanalytical Chemistry

Fobang Liu[1], Senchao Lai[1,2], Kathrin Reinmuth-Selzle[1], Jan Frederik Scheel[1], Janine Fröhlich-Nowoisky[1], Viviane R. Després[3], Thorsten Hoffmann[4], Ulrich Pöschl[1] and Christopher J. Kampf[4,1,*]
[1]Department of Multiphase Chemistry, Max Planck Institute for Chemistry, Mainz, Germany
[2]College of Environment and Energy, South China University of Technology, Guangzhou, China
[3]Institute of General Botany, Johannes Gutenberg University, Mainz, Germany
[4]Institute for Inorganic and Analytical Chemistry, Johannes Gutenberg University, Mainz, Germany
[*]address correspondence to: Christopher Kampf, email: c.kampf@mpic.de, phone: +49 6131 305 6206

Sample ID (size range)	Protein #	Protein names	Protein IDs (from UniProtKB, www.uniprot.org)	Type of evidence for protein existence (from UniProtKB, www.uniprot.org)	Kingdoms of identified proteins	Phyla of identified proteins	Classes of identified proteins	Orders of identified proteins	Families of identified proteins	Genera of identified proteins	Potentially identified species (#)	*Species* of first Protein ID	Peptides (#)	Razor + unique peptides (#)	Unique peptides (#)	Peptides in F1 (#)	Peptides in F2 (#)	Peptides in F3 (#)	Peptides in F4 (#)	Peptides in F5 (#)	Molecular weight (kDa)	Sequence coverage (%)	PEP	Intensity
**344a (> 3 µm)**	1	Polcalcin Phl p 7	O82040	experimental evidence at protein level	Plant	Streptophyta	Liliopsida	Poales	Poaceae	Phleum	1	*Phleum pratense* (Common timothy)	3	3	2	3					8.7	39.7	1.14E-38	1.53E+08
	2	Heat shock 70 kDa protein	Q01233;Q5B2V1;P53421;Q96W30;O93866;Q00043;Q92260;P40918;P53623;Q01877;P87047;P10591;Q10265;P46587;P18694;O59855;P48720;P41797	experimental evidence at transcript level	Fungi	Ascomycota/ Basidiomycota/ Bastocladiomycota	Sordariomycetes/ Eurotiomycetes/ Saccharomycetes/ Dothideomycetes/ Schizosaccharomycetes/ Pucciniomycetes/ Ustilaginomycetes/ Blastocladiomycetes	Sordariales/ …	Sordariaceae/ …	Neurospora/ Fusarium/ …	19	*Neurospora crassa* (red bread mold)	2	2	2		1	1			70.6	3.7	4.76E-11	1.84E+07
	3	High-molecular weight cobalt-containing nitrile hydratase subunit alpha	P21219	experimental evidence at protein level	Bacteria	Actinobacteria	Actinobacteria	Corynebacteriales	Nocardiaceae	Rhodococcus	1	*Rhodococcus rhodochrous*	2	2	2	1	1				22.8	11.3	3.42E-04	1.81E+07
	4	Ovalbumin	P01012	experimental evidence at protein level	Anmial	Chordata	Aves	Galliformes	Phasianidae	Gallus	1	*Gallus gallus* (Chicken)	6	6	5	6					42.9	24.1	1.27E-95	2.32E+08
	5	Protein S100-A7	Q28050	experimental evidence at protein level	Anmial	Chordata	Mammalia	Artiodactyla	Bovidae	Bos	1	*Bos taurus* (Bovine)	3	3	3	3					11.5	26.7	1.49E-09	4.57E+07
**344b (< 3 µm)**	6	Malate dehydrogenase 1, mitochondrial;Malate dehydrogenase 2, mitochondrial;Malate dehydrogenase, chloroplastic	P83373;B3H269;B0BQN0;A3N1U6;Q9ZP06;Q9LKA3;Q43744;P17783;Q9SN86;P85920;P86074;Q7VP41;O82399;Q42972	experimental evidence at protein level	Plant/ Bacteria	Streptophyta/ Proteobacteria	Magnoliopsida/ Conferopsida/ Liliopsida/ Gammaproteobacteria	Rosales/ Brassicales/ Cucurbitales/ Solanales/ Poales/ Coniferales/ Pasteurellales	Rosaceae/ Brassicaceae/ Benincaseae/ Solanaceae/ Poaceae/ Pinaceae/ Pasteurellaceae	Fragaria/ Arabidopsis/ Brassica/ Citrullus/ Oryza/ Capsicum/ Pseudotsuga/ Actinobacillus/ Haemophilus	14	*Fragaria ananassa* (Strawberry)	3	3	3	2	2	2			35.5	7.1	4.26E-08	2.12E+08
	7	Ribulose bisphosphate carboxylase large chain	P25414;P25413;P93936;Q95694;P92445;P93890;Q33162;P93906;Q36800;P28261;P92463;P28262;P25836;P25829;P92306;P28259;Q9BA49;Q6ENG6;P48684;P11383;P0C512;P0C511;P0C510;A8Y9H8;A6MML5;A1EA16;A9L9A4;P48683;P05698;Q3V526;Q3BAN4;A9LYA9;P51994;Q37227;Q8MD78	experimental evidence at protein level	Plant	Streptophyta	Liliopsida	Poales/ Asparagales/ Arecales/ Acorales/ Alismatales/ Dioscoreales	Poaceae/ Iridaceae/ Aspraragaceae/ Hyacinthaceae/ Arecaceae/ Bromeliaceae/ Acoraceae/ Araceae	Aegilops/ Iris/ Oryza/ Avena/ Triticum/ Lolium/ Hordeum/ ...	35	*Tricium aestivum* (wheat)	3	3	1	3	1	1			47.0	7.6	3.90E-09	3.27E+08
	8	14-3-3-like protein GF14-F	Q06967;Q9SP07;P42653;P29305	experimental evidence at protein level	Plant	Streptophyta	Liliopsida	Poales/ Liliales/ Fabales	Poaceae/ Liliaceae/ Fabaceae	Oryza/ Lilium/ Vicia/ Hordeum	4	*Oryza sativa subsp. japonica* (Rice)	3	3	1	2		2			29.2	11.5	6.10E-10	4.83E+07
	9	Hydrophobic seed protein	P24337	experimental evidence at protein level	Plant	Streptophyta	Magnoliopsida	Fabales	Fabaceae	Glycine	1	*Glycine max* (Soybean)	2	2	2	2					8.4	25	2.77E-14	6.21E+08
	10	Beta-conglycinin, alpha chain	P13916	experimental evidence at protein level	Plant	Streptophyta	Magnoliopsida	Fabales	Fabaceae	Glycine	1	*Glycine max* (Soybean)	2	2	1	1	1				70.3	3.5	2.50E-06	1.22E+07
	11	Major pollen allergen Lol p 5a	Q40240	experimental evidence at protein level	Plant	Streptophyta	Liliopsida	Poales	Poaceae	Lolium	1	*Lolium perenne* (Perennial ryegrass)	2	2	1			2			30.9	5.5	1.82E-04	1.11E+07
	12	ATP synthase subunit beta, mitochondrial	P00830;P49376;P85446;P23704	experimental evidence at protein level	Fungi	Ascomycota	Saccharomycetes	Saccharomycetales	Saccharomycetaceae	Saccharomyces/ Kluyveromyces/ Penicillium/ Neurospora	4	*Saccharomyces cerevisiae* (baker's yeast)	7	7	1	5	3	6	3	1	54.8	19.4	9.88E-79	1.56E+09
	13	Eukaryotic translation initiation factor 5A-1	P23301;O94083;P38672	experimental evidence at protein level	Fungi	Ascomycota	Saccharomycetes	Saccharomycetales	Saccharomycetaceae	Saccharomyces	3	*Saccharomyces cerevisiae* (baker's yeast)	2	2	2		2				17.1	8.3	2.30E-06	2.77E+07
	14	Elongation factor 2	Q6BJ25;Q875S0;A5DI11;Q874B9;Q754C8;Q6CPQ9;P32324	experimental evidence at protein level	Fungi	Ascomycota	Saccharomycetes	Saccharomycetales	Saccharomycetaceae	Debaryomyces/ Lachancea/ Meyerozyma/ Komagataella/ Eremothecium/ Kluyveromyces/ Saccharomyces	7	*Saccharomyces cerevisiae* (Baker's yeast)	3	3	1	3	1				93.2	4	3.65E-09	3.58E+07
	15	Histone H2B	P78567	experimental evidence at transcript level	Fungi	Basidiomycota	Agaricomycetes	Agaricales	Agaricaceaea	Agaricus	1	*Agaricus bisporus* (White button mushroom)	2	2	1	1	1				15.2	17.5	6.22E-08	3.24E+07
	16	Small COPII coat GTPase SAR1	P0CR31;P0CR30;Q0UKC0;Q877B9;Q6FUZ9;Q5EMZ6;Q5BGB9;Q4WJS7;Q0CUN7;P78976;P0C951;P0C950;P0C583;A1D4D1;A1CRG9;Q9P4C8;Q755D7;Q6CWR7;Q6CB54;Q6BVA7;Q59S78;Q2HA55;P20606;A5E5G3;A5DR82;A3LTA2	protein inferred from homology	Fungi	Basidiomycota/ Ascomycota	Tremellomycetes/ Dothideomycetes/ Eurotiomycetes/ Saccharomycetes/ Sordariomycetes	Tremellales/ Pleosporales/ Eurotiales/ Saccharomycetales/ Hypocreales/ Sordariales	Tremellaceae/ Phaeosphaeriaceae/ Aspergillaceae/ Saccharomycetaceae/ Hypocreaceae/ Sordariaceae/ Phaffomycetaceae/ Dipodascaceae/ Debaryomycetaceae/ Chaetomiaceae	Cryptococcus/ Aspergillus/ Emericella/ Neosartorya/ Trichoderma/ Neurospora/ Debaryomyces/ Saccharomyces/ Lodderomyces/ Meyerozyma/ Scheffersomyces/ …	26	*Cryptococcus neoformans*	4	4	2	4					21.4	22.8	1.73E-25	6.48E+07
	17	Ras-related protein Rab-11A	P36412;Q5ZJN2;Q5R9M7;Q52NJ1;Q2TA29;P62494;P62493;P62492;P62491;P62490;Q3MHP2;Q15907;P46638;P22129;O35509	experimental evidence at protein level	Amoebozoa/ Animal	Chordata	Mycetozoa/ Aves/ Mammalia	Dictyosteliida/ Galliformes/ …	…	Dictyostelium/ Gallus/ Sus/ Bos/ Rattus/ Mus/ Homo/ Canis/ …	15	*Dictyostelium discoideum* (slime mold)	2	2	2		2				24.0	10.7	2.83E-04	1.75E+07
	18	40S ribosomal protein S14	P19115	protein inferred from homology	Fungi	Ascomycota	Sordariomycetes	Sordariales	Sordariaceae	Neurospora	1	*Neurospora crassa* (red bread mold)	2	2	1	2					16.0	18.7	2.95E-09	6.31E+07
	19	40S ribosomal protein S5	Q7RVI1	protein inferred from homology	Fungi	Ascomycota	Sordariomycetes	Sordariales	Sordariaceae	Neurospora	1	*Neurospora crassa* (red bread mold)	2	2	1	1	2	1			23.7	10.8	5.78E-12	2.88E+07
	20	Heat shock 70 kDa protein	Q01233;Q5B2V1;P53421;Q96W30;O93866;Q00043	experimental evidence at transcript level	Fungi	Ascomycota	Sordariomycetes/ Eurotiomycetes/ Saccharomycetales	Sordariales/ Eurotiales/ Saccharomycetales/ Onygenales	Sordariaceae/ Aspergillaceae/ Pichiaceae/ Arthrodermataceae/ Ajellomycetaceae	Neurospora/ Emericella/ Ogataea/ Paracoccidioides/ Trichophyton/ Histoplasma	6	*Neurospora crassa* (red bread mold)	4	4	4	4	2	1			70.6	7.4	4.76E-11	1.20E+08
	21	Nucleoside diphosphate kinase	A9B9E7;A2STK8	protein inferred from homology	Bacteria/ Archaea	Cyanobacteria/ Euryarchaeota	Methanomicrobia	Prochlorales/ Methanomicrobiales	Prochloraceae/ Methanocorpusculaceae	Prochlorococcus/ Methanocorpusculum	2	*Prochlorococcus marinus*	2	2	2	2	1				16.7	13.9	1.84E-09	7.32E+06
	22	Histone H3-like 1;Histone H3.3-like type 2;Histone H3.2;Histone H3-like 5;Putative histone H3.3-like type 3;Histone H3.3 type 2;Histone H3.3;Histone H3.1;Histone H3.1t;Histone H3.3 type 1;Histone H3;Histone H3.1/H3.2;Histone H3.3C;Histone H3-like 2	Q9U7D1;Q9FX60;Q76MV0;Q71T45;Q6LCK1;Q6LBE3;Q5MYA4;Q2RAD9;Q27532;P69248;P69246;P68430;P68429;P68428;P68427;P59226;P08903;A2Y533;Q9FKQ3;P81198;P81201;P81200;P81199;P81197;P81196;P81195;Q27489;Q7XYZ0;Q55BP0;Q55BN9;Q402E1;Q22RG7;P69150;P69149;P41353;P15512;Q9FXI7;O15819;Q9MBF6	experimental evidence at transcript level	Amoebozoa/ Bacteria/ Fungi/ Animal/ Plant	…	…	…	…	…	39	*Mastigamoeba balamuthi*	2	2	2	2	1				15.2	14.8	1.10E-04	1.92E+08
	23	ATP synthase subunit alpha, mitochondrial	Q9XXK1;P37211;C6E9F3;B9LZ86;B5YI22;B5EFI9;B3EA03;A5G9D6;A1ALL5;Q74GY2;Q39Q54;Q2N8Z5;A5V3X3	experimental evidence at protein level	Animal/ Fungi/ Bacteria	Nematoda/ Ascomycota/ Proteobacteria	Chromadorea/ Sordariomycetes/ Deltaproteobacteria	Rhabditida/ Sordariales/ …	Rhabditidae/ Sordariaceae/ …	Caenorhabditis/ Neurospora/ …	13	*Caenorhabditis elegans*	2	2	1	2					57.8	5	1.50E-05	6.16E+07
	24	Selenium-binding protein 2;Selenium-binding protein 1	Q63836;P17563	experimental evidence at protein level	Animal	Chordata	Mammalia	Rodentia	Muridae	Mus	2	*Mus musculus* (Mouse)	2	2	2	1	2				52.6	2.5	1.04E-05	3.70E+08
	25	Latherin	P82615	experimental evidence at protein level	Animal	Chordata	Mammalia	Perissodactyla	Equidae	Equus	1	*Equus caballus* (Horse)	2	2	1	1	1	1			24.7	10.5	3.68E-13	2.71E+07
	26	Ovalbumin	P01012	experimental evidence at protein level	Anmial	Chordata	Aves	Galliformes	Phasianidae	Gallus	1	*Gallus gallus* (Chicken)	2	2	2	1		1			42.9	9.1	1.27E-95	3.95E+07
**Mz02c (TSP)**	27	Beta-conglycinin, alpha chain	P13916	experimental evidence at protein level	Plant	Streptophyta	Magnoliopsida	Fabales	Fabaceae	Glycine	1	*Glycine max* (Soybean)	9	9	6		2	9	4		70.3	16.7	0.00E+00	4.38E+08
	28	Hydrophobic seed protein	P24337	experimental evidence at protein level	Plant	Streptophyta	Magnoliopsida	Fabales	Fabaceae	Glycine	1	*Glycine max* (Soybean)	3	3	3	2	1			1	8.4	37.5	1.09E-262	2.99E+09
	29	Glycinin G4	P02858	experimental evidence at protein level	Plant	Streptophyta	Magnoliopsida	Fabales	Fabaceae	Glycine	1	*Glycine max* (Soybean)	2	2	2	1	2	1	1		63.6	6	4.70E-230	4.50E+08
	30	Glycinin G2	P04405	experimental evidence at protein level	Plant	Streptophyta	Magnoliopsida	Fabales	Fabaceae	Glycine	1	*Glycine max* (Soybean)	7	7	4	1	5	4			54.4	21.2	9.30E-125	2.64E+08
	31	Beta-conglycinin, alpha' chain	P11827	experimental evidence at transcript level	Plant	Streptophyta	Magnoliopsida	Fabales	Fabaceae	Glycine	1	*Glycine max* (Soybean)	4	4	2			4			74.3	7.2	1.67E-114	8.49E+06
	32	Beta-conglycinin, beta chain	P25974	experimental evidence at protein level	Plant	Streptophyta	Magnoliopsida	Fabales	Fabaceae	Glycine	1	*Glycine max* (Soybean)	4	4	3	1		3			50.6	11.4	2.49E-110	1.83E+07
	33	Trypsin inhibitor A	P01070;P85278;P01071;P25272	experimental evidence at protein level	Plant	Streptophyta	Magnoliopsida/ Liliopsida	Fabales/ Zingiberales	Fabaceae/ Zingiberaceae	Glycine/ Curcuma	4	*Glycine max* (Soybean)	3	3	3	3					24.0	14.4	7.17E-10	4.68E+07
	34	Glycinin G1	P04776	experimental evidence at protein level	Plant	Streptophyta	Magnoliopsida	Fabales	Fabaceae	Glycine	1	*Glycine max* (Soybean)	7	4	4	4	5	3	1		55.7	16.2	1.98E-41	8.59E+08
	35	P24 oleosin isoform B	P29531	experimental evidence at transcript level	Plant	Streptophyta	Magnoliopsida	Fabales	Fabaceae	Glycine	1	*Glycine max* (Soybean)	2	2	1	1	2				23.4	13.5	5.32E-06	1.60E+08
	36	Major pollen allergen Lol p 5b	Q40237	experimental evidence at protein level	Plant	Streptophyta	Liliopsida	Poales	Poaceae	Lolium	1	*Lolium perenne* (perennial ryegrass)	5	5	5			5			33.8	21.8	1.76E-124	3.07E+08
	37	Major pollen allergen Lol p 5a	Q40240	experimental evidence at protein level	Plant	Streptophyta	Liliopsida	Poales	Poaceae	Lolium	1	*Lolium perenne* (Perennial ryegrass)	6	6	6		6	2			30.9	26.4	9.96E-16	3.42E+08
	38	Pollen allergen Lol p 2-A	P14947	experimental evidence at protein level	Plant	Streptophyta	Liliopsida	Poales	Poaceae	Lolium	1	*Lolium perenne* (Perennial ryegrass)	3	3	1	3					10.9	36.1	3.72E-09	1.70E+08
	39	Pollen allergen Dac g 3	P93124	experimental evidence at protein level	Plant	Streptophyta	Liliopsida	Poales	Poaceae	Dactylis	1	*Dactylis glomerata* (Orchard grass)	2	2	1	2					10.9	27.1	8.46E-15	2.96E+08
	40	Pollen allergen Phl p 5a	Q40962	experimental evidence at protein level	Plant	Streptophyta	Liliopsida	Poales	Poaceae	Phleum	1	*Phleum pratense* (Common timothy)	3	3	2		1	3			28.5	15	1.40E-12	1.03E+08
	41	Polcalcin Phl p 7	O82040	experimental evidence at protein level	Plant	Streptophyta	Liliopsida	Poales	Poaceae	Phleum	1	*Phleum pratense* (Common timothy)	2	2	1	2					8.7	29.5	4.63E-05	8.62E+07
	42	Calmodulin-5	Q682T9;Q39752;Q7Y052;Q03509;P93087;P62202;P62201;P62200;P62199;P59220;P48976;P0DH98;P0DH97;P27164;Q7DMP0;Q7DMN9;Q6F332;P93171;P41040;P17928;P0DH96;P0DH95;P04464;P04353;A2Y609	experimental evidence at protein level	Plant	Streptophyta	Magnoliopsida/ Liliopsida	Brassicales/ Poales / Fagales/ …	Brassicaceae/ Poaceae/ Fagaceae/ …	Arabidopsis/ Zea/ Fagus/ …	25	*Arabidopsis thaliana* (Mouse-ear cress)	2	2	1	1	2				16.8	17.6	2.48E-11	1.19E+08
	43	Coatomer subunit gamma	Q6Z382	experimental evidence at transcript level	Plant	Streptophyta	Liliopsida	Poales/ Liliales/ Fabales	Poaceae/ Liliaceae/ Fabaceae	Oryza/ Lilium/ Vicia/ Hordeum	1	*Oryza sativa subsp. japonica* (Rice)	2	2	1				2		98.6	3.7	2.05E-10	2.45E+07
	44	ATP synthase subunit beta	Q01859;P19023;P17614;P85088;P29685;P37399;P83484;P83483;Q9C5A9;P38482	experimental evidence at protein level	Plant	Streptophyta	Liliopsida/ Magnoliopsida	Poales/ Solanales/ Malpighiales/ Vitales/ Apiales/ Brassicales/ Chlamydomonadales	Poaceae/ Solanaceae/ Euphorbiaceae/ Vitaceae/ Apiaceae/ Brassicaceae/ Chlamydomonadaceae	Oryza/ Zea/ Nicotiana/ Hevea/ Vitis/ Daucus/ Arabidopsis / Chlamydomonas	10	*Oryza sativa subsp. japonica* (Rice)	5	4	3	1		4			58.9	13.4	7.97E-53	1.40E+08
	45	Glyceraldehyde-3-phosphate dehydrogenase 2	P08477;Q7FAH2;Q6K5G8;Q43247;Q0J8A4;Q09054;P26520;P26517;P25861;P08735;A2YQT7;Q9FX54;P34922;P34921;P25858;P04796;P34783;P09094;Q6FPW3;Q6BMK0;P26519;Q42671;P17878;P26521;Q6CCU7;P26518	experimental evidence at transcript level	Plant/ Fungi	Streptophyta/ Ascomycota	Liliopsida/ Magnoliopsida/ Saccharomycetes	Poales/ Solanales/ Lamiales/ ... Saccharomycetales	Poaceae/ Solanaceae/ Plantaginaceae/ … Saccharomycetaceae/ Debaryomycetaceae/ Dipodascaceae	Hordeum/ Zea/ Petunia/ Antirrhinum/ … Nakaseomyces/ Debaryomyces/ Yarrowia	26	*Hordeum vulgare* (Barley)	2	2	2	1	2	2			33.2	9.5	4.02E-06	3.00E+08
	46	14-3-3-like protein A	P29305	experimental evidence at transcript level	Plant	Streptophyta	Liliopsida	Poales	Poaceae	Hordeum	1	*Hordeum vulgare* (Barley)	2	2	2		2	1			29.4	11.1	1.82E-08	7.08E+07
	47	ATP synthase subunit alpha	P37211	protein inferred from homology	Fungi	Ascomycota	Sordariomycetes	Sordariales	Sordariaceae	Neurospora	1	*Neurospora crassa*	2	2	2			2			59.5	4.9	4.99E-11	7.88E+07
	48	40S ribosomal protein S14	P19115	protein inferred from homology	Fungi	Ascomycota	Sordariomycetes	Sordariales	Sordariaceae	Neurospora	1	*Neurospora crassa*	4	4	2	4					16.0	34.7	1.34E-10	2.17E+08
	49	Superoxide dismutase [Cu-Zn]	P07509	experimental evidence at protein level	Fungi	Ascomycota	Sordariomycetes	Sordariales	Sordariaceae	Neurospora	1	*Neurospora crassa*	2	2	1	2					15.9	13	3.46E-04	1.50E+08
	50	Glyceraldehyde-3-phosphate dehydrogenase	Q9HGY7;Q8X1X3;Q00640;Q5RAB4;P04406;Q12552;Q9P8C0;Q9HFX1;Q8J1H3;P53430;P29497;P28844;P19089;Q96US8;Q8WZN0;P54118;P35143;P80534	experimental evidence at transcript level	Fungi/ Animal	Ascomycota/ Chordata	Eurotiomycetes/ Leotiomycetes/ Dothideomycetes/ Sordariomycetes/ Mammalia	Eurotiales/ Erysiphales/ Pleosporales/ Sordariales/ ... Primates/ Rodentia	Aspergillaceae/ Erysiphaceae/ Phaeosphaeriaceae/ Sordariaceae/ … Hominidae/ Dipodidae	Aspergillus/ Blumeria/ Parastagonospora/ Sordaria/ … Pongo/ Homo/ Jaculus	18	*Aspergillus oryzae*	2	2	2	1	2	2			36.2	8.6	1.57E-254	1.15E+08

51	Calmodulin	Q9UWF0;P61861;P61860;P61859;P60206;<br>P60205;P60204;P07463	protein inferred from homology	Fungi/<br>Alveolata	Ascomycota/<br>Ciliophora	Sordariomycetes/<br>Eurotiomycetes/<br>Oligohymenophorea	Magnaporthales/<br>Glomerellales/<br>Sordariales/<br>Onygenales/ Eurotiales/<br>Peniculida	Magnaporthaceae/<br>Glomerellaceae/<br>Sordariaceae/<br>Ajellomycetaceae/<br>Aspergillaceae/ Parameciidae	Magnaporthe/<br>Colletotrichum/<br>Neurospora/<br>Histoplasma/<br>Aspergillus/<br>Paramecium	8	*Magnaporthe oryzae*	2	2	2	2	1			17.0	16.1	6.29E-32	9.16E+07
52	ADP,ATP carrier protein	Q09188	experimental evidence at transcript level	Fungi	Ascomycota	Schizosaccharomycetes	Schizosaccharomycetales	Schizosaccharomycetaceae	Schizosaccharomyces	1	*Schizosaccharomyces pombe*<br>(Fission yeast)	3	3	1	2	1		1	35.0	9	2.04E-09	3.71E+07
53	Histone H4	P09322;Q8NIG3;Q8J1L3;Q75AX1;Q757K<br>0;P91890;P90516;Q8T7J8;Q6ZXX3;P0230<br>9	experimental evidence at protein level	Fungi/ Animal/<br>Alveolata	Ascomycota/<br>Basidiomycota/<br>Arthropoda/<br>Ciliophora/<br>Apicomplexa	Schizosaccharomycetes/<br>Saccharomycetes/<br>Ustilaginomycetes/<br>Insecta/<br>Heterotrichea/<br>Coccidia	Schizosaccharomycetales/<br>Saccharomycetales/<br>Ustilaginales/<br>Hymenoptera/<br>Heterotrichida/<br>Eucoccidiorida	Schizosaccharomycetaceae/<br>Saccharomycetaceae/<br>Trichomonascaceae/<br>Ustilaginales/<br>Trichogrammatidae/<br>Blepharismidae/<br>Eimeriidae	Schizosaccharomyces/<br>Blastobotrys/<br>Ustilago/ …<br>Trichogramma/<br>Blepharisma/<br>Eimeria	10	*Schizosaccharomyces pombe*<br>(Fission yeast)	2	2	1	2	1			11.4	29.1	9.58E-98	3.12E+08
54	Enolase	P42040	experimental evidence at protein level	Fungi	Ascomycota	Dothideomycetes	Capnodiales	Cladosporiaceae	Cladosporium	1	*Davidiella tassiana*	2	2	2		1	2		47.5	6.8	3.30E-129	1.41E+08
55	Trihydroxynaphthalene reductase	P87025	protein inferred from homology	Fungi	Ascomycota	Sordariomycetes	Glomerellales	Glomerellaceae	Colletotrichum	1	*Colletotrichum orbiculare*	2	2	2		1	2		29.8	7.8	9.45E-08	8.65E+07
56	ATP-dependent RNA helicase eIF4A	Q0UU86;A4QVP2;Q7RV88;A6R3R5;Q2H<br>FP1;A7EGL7;A6RJ45;A1CJT5;P47943;Q6<br>CDV4;P10081;A6ZQJ1;Q6FQQ6;A7TK55;<br>A5DB98;Q0CXD0;Q6BRN4;P87206;A5D<br>VM3;A3GFI4;Q5B948;Q1DQ20;A2QEN5;<br>Q2UPY3	protein inferred from homology	Fungi	Ascomycota	Dothideomycetes/<br>Sordariomycetes/<br>Eurotiomycetes/<br>Leotiomycetes/<br>Schizosaccharomycetes/<br>Saccharomycetes	Pleosporales/<br>Magnaporthales/<br>Onygenales/ …	Phaeosphaeriaceae/<br>Magnaporthaceae/<br>Ajellomycetaceae/ …	Parastagonospora/<br>Magnaporthe/<br>Histoplasma/ …	24	*Phaeosphaeria nodorum*<br>(Glume blotch fungus)	3	3	2	1	2	2		44.5	8.8	5.66E-07	1.45E+08
57	Malate dehydrogenase 1	B3H269;B0BQN0;A3N1U6;P83373;Q9ZP<br>06;Q9LKA3;Q43744;P17783;Q9SN86;P85<br>920;P86074;Q7VP41	protein inferred from homology for bacteria, experimenta evidence at protein/transcript level for plant protein	Bacteria/ Plant	Proteobacteria/<br>Streptophyta	Gammaproteobacteria/<br>Magnoliopsida/ Pinopsida	Pasteurellales/<br>Rosales/<br>Brassicales/<br>Cucurbitales/ Pinales/<br>Solanales	Pasteurellaceae/<br>Rosaceae/<br>Brassicaceae/<br>Cucurbitaceae/<br>Pinaceae/<br>Solanaceae	Actinobacillus/<br>Haemophilus/<br>Fragaria/<br>Arabidopsis/<br>Brassica/<br>Citrullus/ ...	12	*Actinobacillus pleuropneumoniae serotype 7*	2	2	2	1	1	2		33.4	3.8	8.75E-103	9.27E+07
58	Ovalbumin	P01012	experimental evidence at protein level	Anmial	Chordata	Aves	Galliformes	Phasianidae	Gallus	1	*Gallus gallus*<br>(Chicken)	2	2	1		1	1		42.9	8.5	1.09E-05	4.27E+07
59	Latherin	P82615	experimental evidence at protein level	Animal	Chordata	Mammalia	Perissodactyla	Equidae	Equus	1	*Equus caballus*<br>(Horse)	3	3	1	3	2			24.7	15.8	2.10E-27	1.36E+08

assignement of taxonomic rank ambigous at kingdom revel
assignement of taxonomic rank ambigous at phylum level
